# Non-parenchymal TREM-2 protects the liver from immune-mediated hepatocellular damage

**DOI:** 10.1136/gutjnl-2017-314107

**Published:** 2018-01-27

**Authors:** Maria J Perugorria, Aitor Esparza-Baquer, Fiona Oakley, Ibone Labiano, Ana Korosec, Alexander Jais, Jelena Mann, Dina Tiniakos, Alvaro Santos-Laso, Ander Arbelaiz, Riem Gawish, Ana Sampedro, Antonio Fontanellas, Elizabeth Hijona, Raul Jimenez-Agüero, Harald Esterbauer, Dagmar Stoiber, Luis Bujanda, Jesus María Banales, Sylvia Knapp, Omar Sharif, Derek A Mann

**Affiliations:** 1 Newcastle Fibrosis Research Group, Institute of Cellular Medicine, Faculty of Medical Sciences, Newcastle University, Newcastle upon Tyne, UK; 2 Department of Liver and Gastrointestinal Diseases, Biodonostia Research Institute, Donostia University Hospital, University of the Basque Country (UPV-EHU), San Sebastian, Spain; 3 IKERBASQUE, Basque Foundation for Science, Bilbao, Spain; 4 CIBERehd, Instituto de Salud Carlos III, San Sebastián, Spain; 5 CeMM, Research Center for Molecular Medicine of the Austrian Academy of Sciences, Vienna, Austria; 6 Department of Medicine I, Laboratory of Infection Biology, Medical University of Vienna, Vienna, Austria; 7 Department of Laboratory Medicine, Medical University of Vienna, Vienna, Austria; 8 Hepatology Programme, CIMA, University of Navarra, Pamplona, Spain; 9 Institute of Pharmacology, Center of Physiology and Pharmacology, Medical University of Vienna, Vienna, Austria; 10 Ludwig Boltzmann Institute for Cancer Research, Vienna, Austria

**Keywords:** acute liver failure, chronic liver disease, hepatic stellate cell, inflammation, immune-mediated liver damage

## Abstract

**Objective:**

Liver injury impacts hepatic inflammation in part via Toll-like receptor (TLR) signalling. Triggering receptor expressed on myeloid cells 2 (TREM-2) modulates TLR4-mediated inflammation in bone marrow (BM)-derived macrophages but its function in liver injury is unknown. Here we hypothesised that the anti-inflammatory effects of TREM-2 on TLR signalling may limit hepatic injury.

**Design:**

TREM-2 expression was analysed in livers of humans with various forms of liver injury compared with control individuals. Acute and chronic liver injury models were performed in wild type and *Trem-2^-/-^* mice. Primary liver cells from both genotypes of mice were isolated for in vitro experiments.

**Results:**

TREM-2 was expressed on non-parenchymal hepatic cells and induced during liver injury in mice and man. Mice lacking TREM-2 exhibited heightened liver damage and inflammation during acute and repetitive carbon tetrachloride and acetaminophen (APAP) intoxication, the latter of which TREM-2 deficiency was remarkably associated with worsened survival. Liver damage in *Trem-2^-/-^* mice following chronic injury and APAP challenge was associated with elevated hepatic lipid peroxidation and macrophage content. BM transplantation experiments and cellular reactive oxygen species assays revealed effects of TREM-2 in the context of chronic injury depended on both immune and resident TREM-2 expression. Consistent with effects of TREM-2 on inflammation-associated injury, primary hepatic macrophages and hepatic stellate cells lacking TREM-2 exhibited augmented TLR4-driven proinflammatory responses.

**Conclusion:**

Our data indicate that by acting as a natural brake on inflammation during hepatocellular injury, TREM-2 is a critical regulator of diverse types of hepatotoxic injury.

**Significance of this study**

**What is already known on this subject?**

- Increasing evidence suggests the gut-liver axis and innate immunity play a significant role in liver injury. Impaired intestinal permeability may promote liver inflammation due to the translocation of bacterial components into the liver.
- The consequent hepatic inflammatory response is in part mediated by toll-like receptors (TLRs). Fine tuning of TLR-driven inflammation during liver injury is key. The liver requires mechanisms to control the intensity and duration of TLR-driven cytokine production, which can contribute to the pathogenesis of acute and chronic liver disease.
- Triggering receptor expressed on myeloid cells 2 (TREM-2) has been proposed to attenuate TLR4-mediated inflammation and to be important in injury responses in colon and brain.

**What are the new findings?**

- TREM-2 is expressed on non-parenchymal liver cells and is upregulated during diverse forms of liver injury in humans and mice.
- *Trem-2^-/-^* mice display increased liver damage and inflammation during acute and chronic carbon tetrachloride treatment and acetaminophen (APAP) intoxication. Effects of TREM-2 in the context of chronic liver injury are dependent on both liver resident and infiltrating immune cells.
- TREM-2 promotes survival during drug-induced APAP challenge.
- Liver injury in *Trem-2^-/-^* mice is associated with elevated levels of reactive oxygen species and lipid peroxidation.
- In line with TREM-2 effects on inflammation and injury, Kupffer cells and hepatic stellate cells isolated from *Trem-2^-/-^* mice exhibit increased proinflammatory cytokine and chemokine production upon TLR4 stimulation.

**Significance of this study**

**How might it impact on clinical practice in the foreseeable future?**

- Strategies that aim to therapeutically activate this receptor may provide a novel route towards suppressing inflammation caused by toxin-induced hepatocellular injuries.

## Introduction

Toll-like receptors (TLRs) are pattern recognition receptors that recognise conserved microbial molecules. A variety of TLRs are expressed on different liver cell types, including hepatocytes, hepatic stellate cells (HSCs) and resident Kupffer cells (KCs), with the latter cell type being located in the hepatic sinusoids and the first cells to encounter gut derived danger signals, such as lipopolysaccharide (LPS).^1–3^ The crucial role of TLR signalling within the liver is highlighted by studies showing that *Tlr4* ^-/-^ mice are protected from various forms of liver injury.^4 5^ Illustrating the critical role of the fine tuning of TLR4-driven inflammation during liver injury, interleukin 1 receptor-associated kinase-M (IRAK-M) and A20, prototypical negative regulators of TLR4 signalling, protect from alcohol-induced and ischaemia/reperfusion liver injury, respectively.^6 7^

The triggering receptor expressed on myeloid cells 2 (TREM-2) is a relatively recent described regulator of TLR-mediated inflammatory responses. Similar to IRAK-M and A20, TREM-2 acts to dampen TLR-driven cytokine responses within bone marrow derived macrophages (BMDM).^8^ This is in contrast to TREM-1 which augments TLR-induced inflammation.^9^ TREM-1 and TREM-2 both signal via the ITAM motif of the adaptor protein DNAX-activation protein 12, which becomes tyrosine phosphorylated leading to increases in intracellular calcium and extracellular regulated kinase (ERK1/2) phosphorylation.^9–11^ TREM-1 is expressed on KCs and HSCs and is important in promoting inflammation-driven hepatocellular carcinoma and diethylnitrosamine liver injury.^12^ TREM-2 has been shown to be expressed on KCs where it serves to control liver stage malarial infection.^13 14^ Given that TREM-2 has been proposed to attenuate TLR4-mediated inflammation and has previously been shown to be important in injury responses in the colon and brain, we undertook this study to examine if it could play a role in liver injury and inflammation.^11 15–17^

## Materials and methods

### Patients

To perform gene expression analysis at mRNA level, liver tissue was obtained from control individuals (n=21) and patients with liver cirrhosis of different aetiology (n=23). Patient characteristics and liver injury-related serology are indicated in online supplementary table 1. Tissue samples of normal liver used as controls were obtained from background non-neoplastic tissue of liver resection specimens for colorectal metastasis. The selected sections were located furthest away from the tumour mass lesion. Samples and data from patients included in this study were provided by the Basque Biobank (http://www.biobancovasco.org) and were processed following standard operation procedures with appropriate approval by the Clinical Research Ethics Committees of the Basque Country and Donostia Hospital. An informed consent was obtained from all subjects. Human hepatic myofibroblasts were isolated from livers of adult male patients after surgical resection as approved by the Newcastle and North Tyneside Local Research Ethics Committee, subject to patient consent (10/H0906/41).

10.1136/gutjnl-2017-314107.supp1 Supplementary file 1

### Experimental models of liver injury

Wild type (WT) and *Trem-2* knockout (*Trem-2^-/-^*) male mice (25–30 g) were intraperitoneally injected one time (acute) or two times a week for 8 weeks (chronic) with carbon tetrachloride (CCl_4_) at 2 µl/g body weight (CCl_4_: olive oil at 1:1 (vol:vol) (acute) and 1:3 (vol:vol) (chronic)). For the acetaminophen (APAP) model WT and *Trem-2^-/-^* male mice (25–30 g) were overnight-starved and intraperitoneally injected once with APAP (Sigma-Aldrich) at a concentration of 300 mg/kg or 500 mg/kg dissolved in sterile warm saline. For survival studies, mice were injected with a lethal dose of APAP (750 mg/kg) and survival was monitored every hour for 30 hours, after which it was monitored every day. Animal care and procedures were approved by the Animal Care and Use Committee of the Medical University of Vienna and the Austrian Ministry of Sciences as well as by the Animal Experimentation Ethics Committee (CEEA) of Biodonostia Research Institute and the Newcastle Ethical Review Committee under a UK Home Office project license. *Trem-2* ^-/-^ mice backcrossed onto a >98% C57BL/6 background were obtained from Marco Colonna (Washington University) and generated as previously described.^8^ For all in vivo experiments, we used age-matched (8–10 week old) male *Trem-2^-/-^* mice and WT controls bred at the Medical University of Vienna or Biodonostia Research Institute. For bone marrow (BM) transplantation, *Trem-2* ^-/-^ mice were crossed with mice that ubiquitously express green-fluorescent protein (GFP) on a C57BL/6 background (WT-UbGFP^+^) to generate (*Trem-2* ^-/-^/Ub-GFP^+^) mice.

### Isolation of mouse hepatocytes, KCs, HSCs, BMDMs and treatments

Hepatocytes, KCs and HSCs were isolated from mice and rats as previously described.^18 19^

Additional Materials and Methods are included as online supplementary files.

10.1136/gutjnl-2017-314107.supp2 Supplementary file 2

## Results

### TREM-2 is upregulated during liver injury in mice and humans

**Figure 1:**
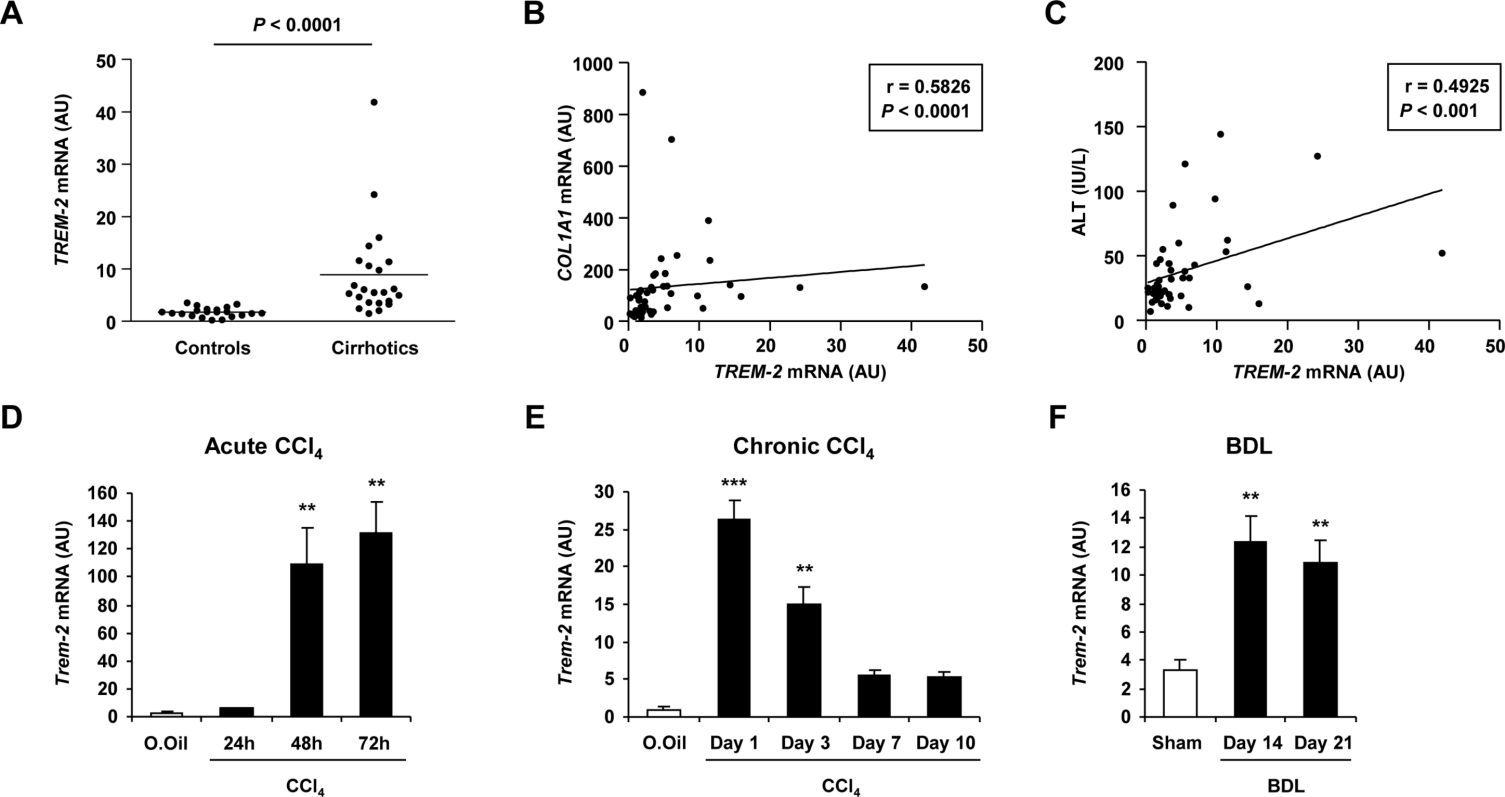
Expression of TREM-2 during human and mouse liver disease. (A) qRT-PCR analysis of *TREM-2* expression in normal human liver (controls) and cirrhotic samples. (B,C) Correlation between *TREM-2* and *COL1A1* levels (B) or ALT (C). n=21 controls and 23 cirrhotic livers. (D) qRT-PCR of *Trem-2* expression in livers of mice after the indicated acute CCl_4_ time points. Olive oil treated mice were used as controls. n=3 mice per condition and time point. (E,F) *Trem-2* mRNA expression in the liver of mice treated with CCl_4_ for 12 weeks and sacrificed 1, 3, 7 or 10 days after the last CCl_4_ injection (E) and 14 or 21 days after BDL (F). n=3–5 (E) and 4–5 mice per time point (F). Statistical analysis used was Mann Whitney test (A) and non-parametric Spearman’s correlation test (B,C). Data represent mean±SEM and **,*** denote a P value of ˂0.01 and ˂0.001, respectively versus olive oil (D,E) or sham (F) determined using one-way analysis of variance, followed by Tukey’s posthoc test. ALT, alanine aminotransferase; BDL, bile duct ligation; CCl_4_, carbon tetrachloride; TREM, triggering receptor expressed on myeloid cells.

We first examined *TREM-2* transcript levels in a cohort of control (n=21) and diseased liver (n=23) as described in online supplementary table 1. By grouping all the cirrhotic livers despite of different disease aetiology (HCV or HBV infection, HCV/HIV coinfection and alcoholic or other aetiology) and comparing these with control liver tissues, we could observe significantly elevated *TREM-2* transcript levels in cirrhotic versus control liver (figure 1A). Correlating these data with levels of markers of hepatocyte injury, fibrosis and inflammation revealed that increases in *TREM-2* expression correlated with collagen levels and with alanine aminotransferase, aspartate aminotransferase and the inflammation markers *IL-8* and *IL-6* (figure 1B,C, online supplementary figure 1), suggesting that increases in TREM-2 may serve to dampen hepatic inflammation and injury. Evaluating TREM-2 expression using immunohistochemistry in an independent cohort of human control liver and cirrhotic liver of variable aetiology (online supplementary table 2), verified that TREM-2 expression was more extensive in diseased compared with control liver (online supplementary figure 2). Mild cytoplasmic TREM-2 immunostaining was observed in monocytes and KCs in control liver, while there were no TREM-2 positive endothelial cells or lymphocytes. TREM-2 immunohistochemical expression in cirrhotic liver was observed in neutrophils, monocytes and macrophages in the fibrous septa, within sinusoids and in parenchymal inflammatory foci. Collagen deposition in fibrous septa was confirmed in cirrhotic versus control liver by Sirius red staining (online supplementary figure 2). Further, *Trem-2* was upregulated during both acute and repetitive CCl_4_ induced injury in mice compared with olive oil controls and augmented *Trem-2* levels occurred during bile duct ligation compared with sham operated mice (figure 1D–F). In the chronic CCl_4_ model, we noted that on cessation of liver damage levels of *Trem-2* transcript declined over a 10-day period of recovery, but remained slightly higher than in controls (figure 1E). We conclude liver injury in mice and man is associated with a substantial induction in the expression of TREM-2 indicating a role for the receptor in hepatic wound repair.

**Figure 2:**
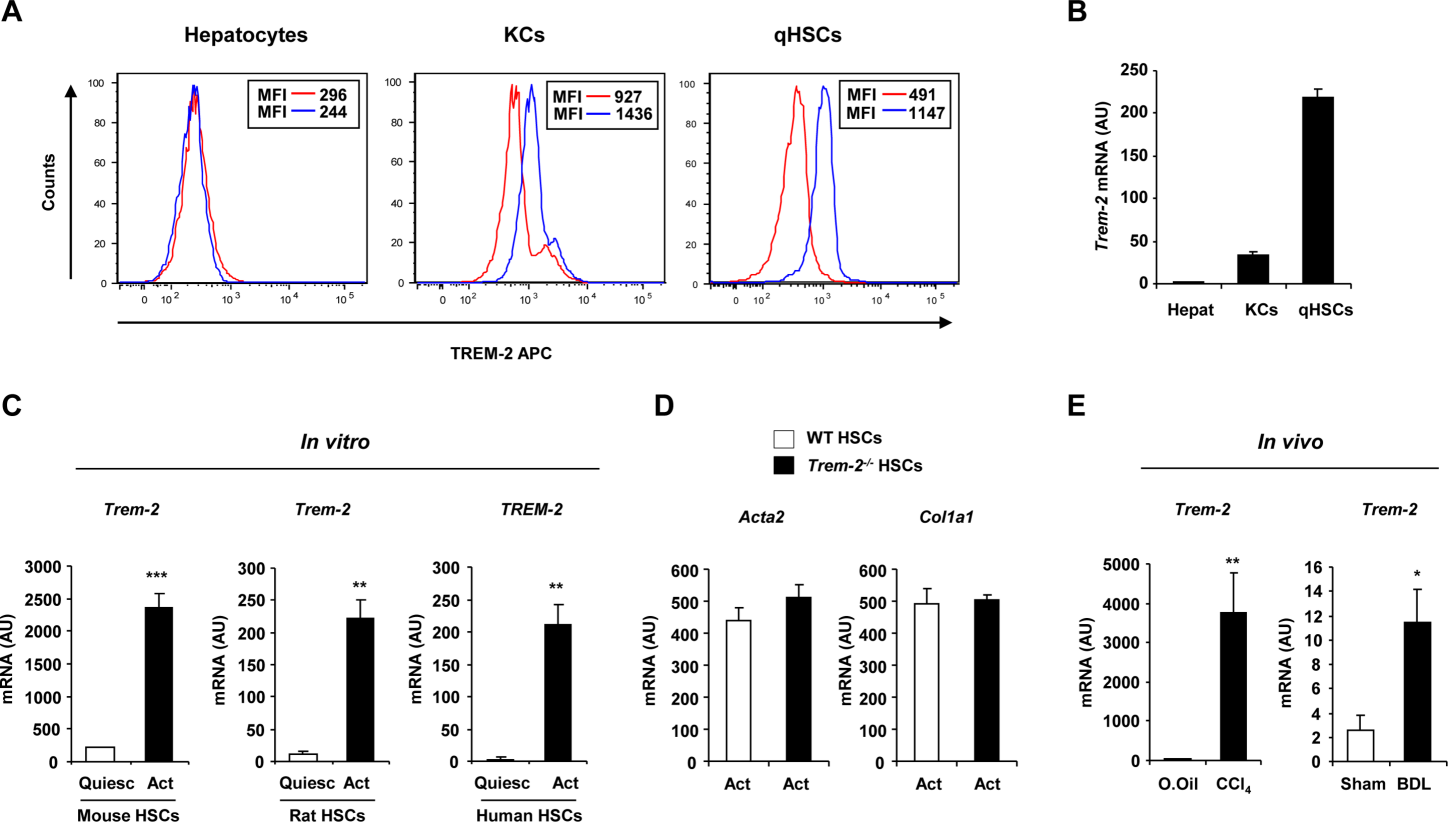
TREM-2 expression in non-parenchymal liver cells and activated HSCs during liver injury. (A) TREM-2 expression in primary mouse hepatocytes, KCs or qHSCs detected by flow cytometry. Red and blue lines depict the isotype and TREM-2 antibodies respectively. (B) qRT-PCR analysis of *Trem-2* expression in various liver cell types. n=10 (hepatocytes), 4 (KCs) and 5 (qHSCs). (C) *TREM-2* expression in mouse, rat and human HSCs during trans-differentiation in vitro at day 1 (quiescent) and day 7 (activated) after the isolation. n=3. (D) Transcript levels of *Acta2* and *Col1a1* in WT and *Trem-2^-/-^* activated mouse HSCs. n=3. (E) *Trem-2* expression in rat HSCs activated in vivo from CCl_4_ and BDL treated rats. n=4–5 per condition. Data represent mean±SEM and *, **, *** denote a P value of ˂0.05, ˂0.01 and ˂0.001, respectively versus quiescent (C) or olive oil or sham (E) and statistical analysis used was unpaired Student’s t-test. Data in (C,E) are representative of two and in (D) is representative of three independent experiments BDL, bile duct ligation; CCl_4_, carbon tetrachloride; HSCs, hepatic stellate cells; KCs, Kupffer cells; qHSC, quiescent HSC; TREM, triggering receptor expressed on myeloid cells; WT, wild type.

### TREM-2 is expressed on non-parenchymal liver cells and is upregulated during HSC activation

Flow cytometry and qRT-PCR for TREM-2 in murine hepatic cells demonstrated expression on KCs and quiescent HSCs, but not on hepatocytes demonstrating that TREM-2 was expressed by non-parenchymal liver cells (figure 2A,B). We next investigated the expression of *Trem-2* during HSC transdifferentiation, which was induced in vitro by culturing freshly isolated murine quiescent HSCs on plastic for 7 days. By doing so, we could observe that *Trem-2* mRNA expression was strongly induced during mouse HSC transdifferentiation in vitro. This response was conserved as *TREM-2* expression during activation in vitro was similarly increased in rat and human HSCs (figure 2C). Examining the activation status of both genotypes of HSCs revealed that activated *Trem-2^-/-^* HSCs displayed similar α-smooth muscle actin (encoded by *Acta2*) and α1(I) procollagen (encoded by *Col1a1*) expression to WT (figure 2D). To address if *Trem-2* was induced during HSC activation in vivo, we isolated HSCs from bile duct ligated or chronic CCl_4_ injured rats. *Trem-2* was significantly induced in HSCs in vivo during liver injury (figure 2E). Together, these data suggested that conserved increases in *TREM-2* expression during HSC activation might play a role during chronic liver injury, a process where HSCs are crucial fibrogenic effector cells and are important mediators of innate immunity that cross-talk with other liver resident and infiltrating cells during hepatocellular damage.^20 21^

### TREM-2 attenuates chronic hepatic inflammation and injury

**Figure 3:**
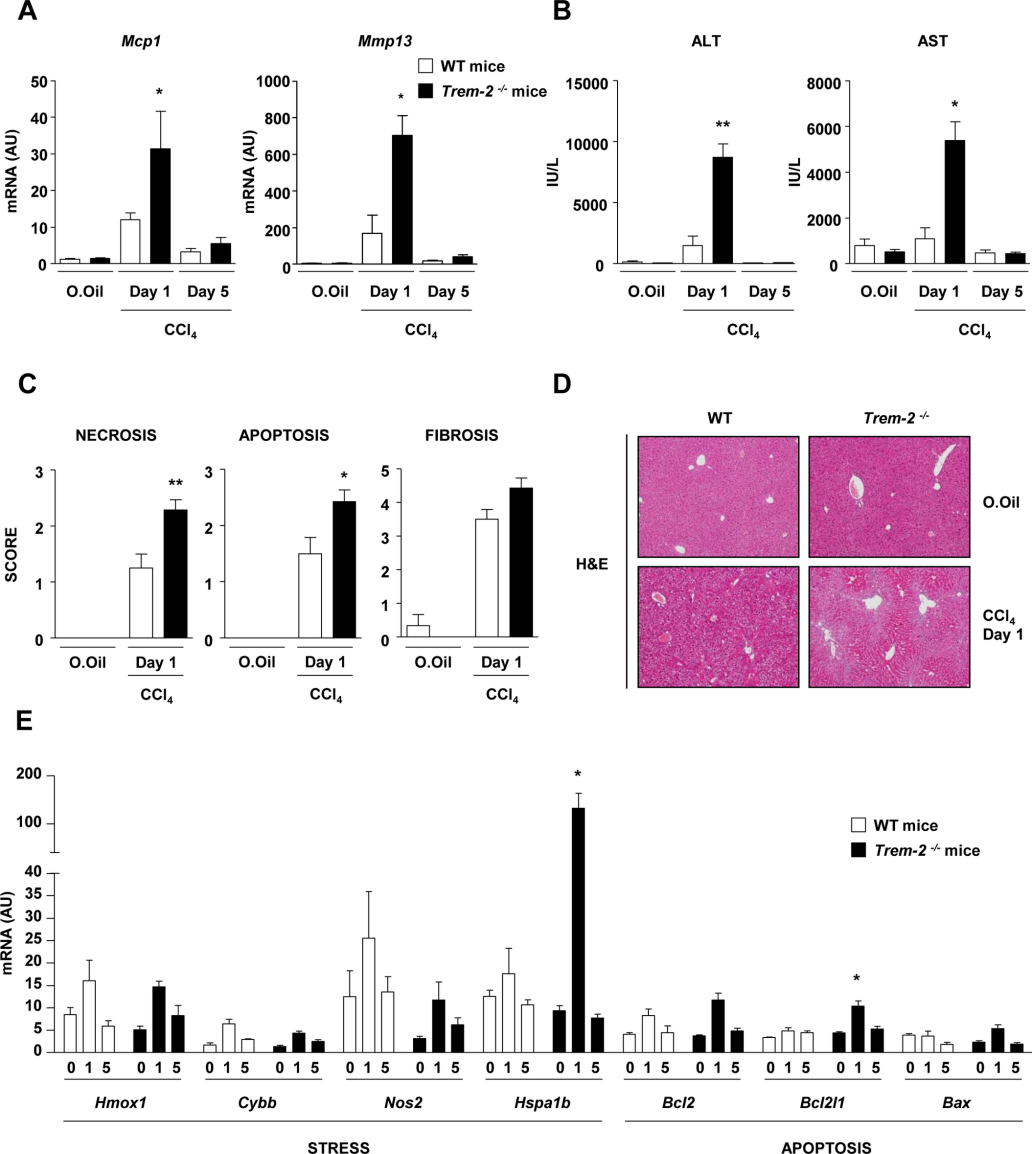
TREM-2 impacts chronic CCl_4_-induced liver injury. (A–E) WT and *Trem-2^-/-^* mice were treated with CCl_4_ for 8 weeks, sacrificed 1 or 5 days after the last CCl_4_ injection and (A) liver *Mcp1*, *Mmp13* (B) ALT and AST levels were determined. (C) Necrosis, apoptosis and fibrosis histology score from WT and *Trem-2^-/-^* mice that were treated with CCl_4_ for 8 weeks and sacrificed 1 day after the last CCl_4_ injection. (D) Representative H&E (Magnification is 10x) from day 1 are depicted. (E) Transcript levels of the indicated genes associated with stress and apoptosis were determined. n=3 mice per genotype (olive oil) and 4–8 mice per genotype (CCl_4_ both time points). Data represent mean±SEM and *, ** denote a P value of ˂0.05 and ˂0.01, respectively versus WT at the same time point (Mann Whitney test). ALT, alanine aminotransferase; AST, aspartate aminotransferase; CCl_4_, carbon tetrachloride; TREM, triggering receptor expressed on myeloid cells; WT, wild type.

To formally investigate a function for TREM-2 in chronic liver disease, we employed a genetic approach comparing damage-induced responses of *Trem2^-/-^* mice with WT controls. We thus performed chronic CCl_4_ injury and first determined differences in hepatic levels of the inflammatory mediator monocyte chemoattractant protein 1 (*Mcp1*). We observed significantly elevated *Mcp1* mRNA in WT at peak injury (day 1 after the final administration of CCl_4_) which declined close to control levels with recovery (day 5 postinjury) (figure 3A). Similarly, as expected, levels of the matrix metalloproteinase-13 (*Mmp13*) transcript were induced with peak injury in WT liver and declined to control levels with recovery (figure 3A). Both *Mcp1* and *Mmp13* were induced at significantly elevated levels in peak injured *Trem-2^-/-^* livers compared with WT, although with recovery no differences were observed between genotypes. Intriguingly we did not observe differences for damage-induced expression of interleukin-6 (*Il6)*, interleukin-1 beta (*Il1b*), tumour necrosis factor-alpha (*Tnf*) or transforming growth factor beta-1 (*Tgfb1*) (online supplementary figure 3). Although at peak injury there was a tendency for higher *Acta2* and *Col1a1* liver transcript levels in *Trem-2^-/-^* mice, Sirius red staining verified that TREM-2 exerted only minor effects on fibrosis (online supplementary figure 4). Augmented inflammation during peak injury in *Trem-2^-/-^* livers was however associated with significantly enhanced transaminase release, suggesting that increased death and damage of hepatocytes occurs in the absence of TREM-2 (figure 3B). In agreement, histology disclosed significantly more necrosis and apoptosis in *Trem-2^-/-^* livers but unremarkable effects of TREM-2 on fibrosis (figure 3C,D). Monitoring transcription of genes associated with cellular stress (Heme oxygenase 1 (*Hmox-1*), cytochrome b-245 heavy chain (*Cybb*), nitric oxide synthase 2 (*Nos2*), Heat-shock family member 70 (*Hspa1b*)) and apoptosis (B-cell CLL Lymphoma 2 (*Bcl-2*), BCL2-like 1 (*Bcl2l1*), BCL-2 associated-X protein (*Bax*)) revealed that these pathways were both operational at peak injury and that levels of the stress-associated marker *Hspa1b* and the antiapoptotic gene *Bcl2l1* were significantly upregulated in *Trem-2^-/-^* compared with WT livers (figure 3E). We conclude that an important function of TREM-2 is to protect against excessive liver injury in the context of iterative hepatotoxic damage.

**Figure 4:**
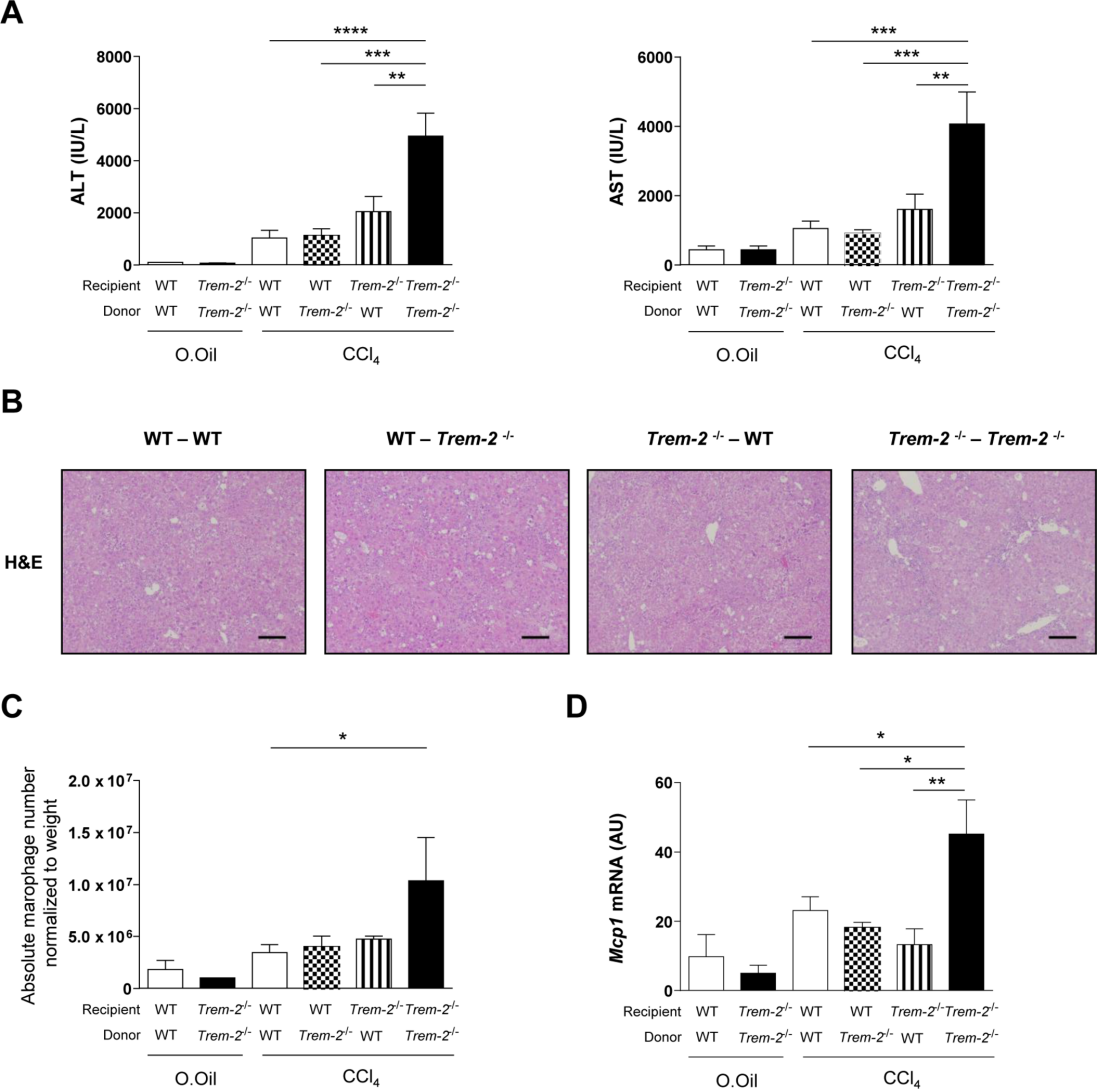
TREM-2 expression within liver resident and infiltrating immune cells is required for dampening of chronic CCl_4_ induced liver injury. (A–D) WT mice reconstituted with WT-GFP^+^bone marrow (WT-WT), *Trem-2^-/-^* mice reconstituted with *Trem-2^-/-^*-GFP^+^bone marrow (*Trem-2^-/-^-Trem-2^-/-^*) or chimeric mice (WT-*Trem-2^-/-^*) and (*Trem-2^-/-^*-WT) were treated with CCl_4_ for 8 weeks and sacrificed 1 day after the last CCl_4_ injection and (A) serum ALT and AST levels, (B) H&E stain of livers (C) total number of macrophages (defined as CD45^+^CD11b^+^Ly6C^+^Ly6G^-^F4/80^+^GFP^+^ cells, (online supplementary figure 5) normalised to liver weight and (D) liver *Mcp1* transcript levels were determined. Data represent mean±SEM and *, **, ***, **** denote a P value of ˂0.05, ˂0.01, ˂0.001 and <0.0001 respectively versus the indicated genotype (one-way analysis of variance, followed by Tukey’s posthoc). n=3 per genotype (olive oil) and 3–5 per genotype (CCl_4_). ALT, alanine aminotransferase; AST, aspartate aminotransferase; CCl_4_, carbon tetrachloride; GFP, green fluorescent protein; TREM, triggering receptor expressed on myeloid cells; WT, wild type.

### Combined action of liver resident and infiltrating cells are required for TREM-2 to dampen chronic injury

To understand the contribution of TREM-2 expression on inflammatory cells versus HSCs to liver damage, we next reconstituted lethally irradiated WT or *Trem-2^-/-^* mice with WT or *Trem-2^-/-^* BM that ubiquitously expresses GFP within both genotypes, to generate four groups of mice. Nearly 100% of blood leucocytes were GFP^+^ within all groups, proving successful transplantation (online supplementary figure 5). Corroborating observations that TREM-2 attenuated chronic liver injury (figure 3B), elevated transaminases were observed in *Trem-2^-/-^* mice reconstituted with *Trem-2^-/-^* BM compared with WT (figure 4A). Although *Trem-2^-/-^* mice reconstituted with WT BM displayed a tendency for more liver damage compared with WT mice transplanted with WT BM, significantly elevated damage was only observed when TREM-2 was deficient within immune and resident cells (such as HSCs), demonstrating TREM-2 protects from immune-mediated hepatocellular damage via its combined functions in these cell types. These observations were confirmed by histology (figure 4B). Monitoring hepatic immune cell influx revealed equal numbers of recruited neutrophils between the groups of mice during chronic injury, while recruited monocyte derived macrophages were significantly elevated in *Trem-2^-/-^* mice reconstituted with *Trem-2^-/-^* BM, which correlated with elevated levels of *Mcp1* (figure 4C,D, online supplementary figures 5 and 6). Together, these observations further support the concept that in the context of chronic CCl_4_ injury, TREM-2 dampens liver injury and indicates that these effects are dependent on both resident and infiltrating immune cells.

**Figure 5:**
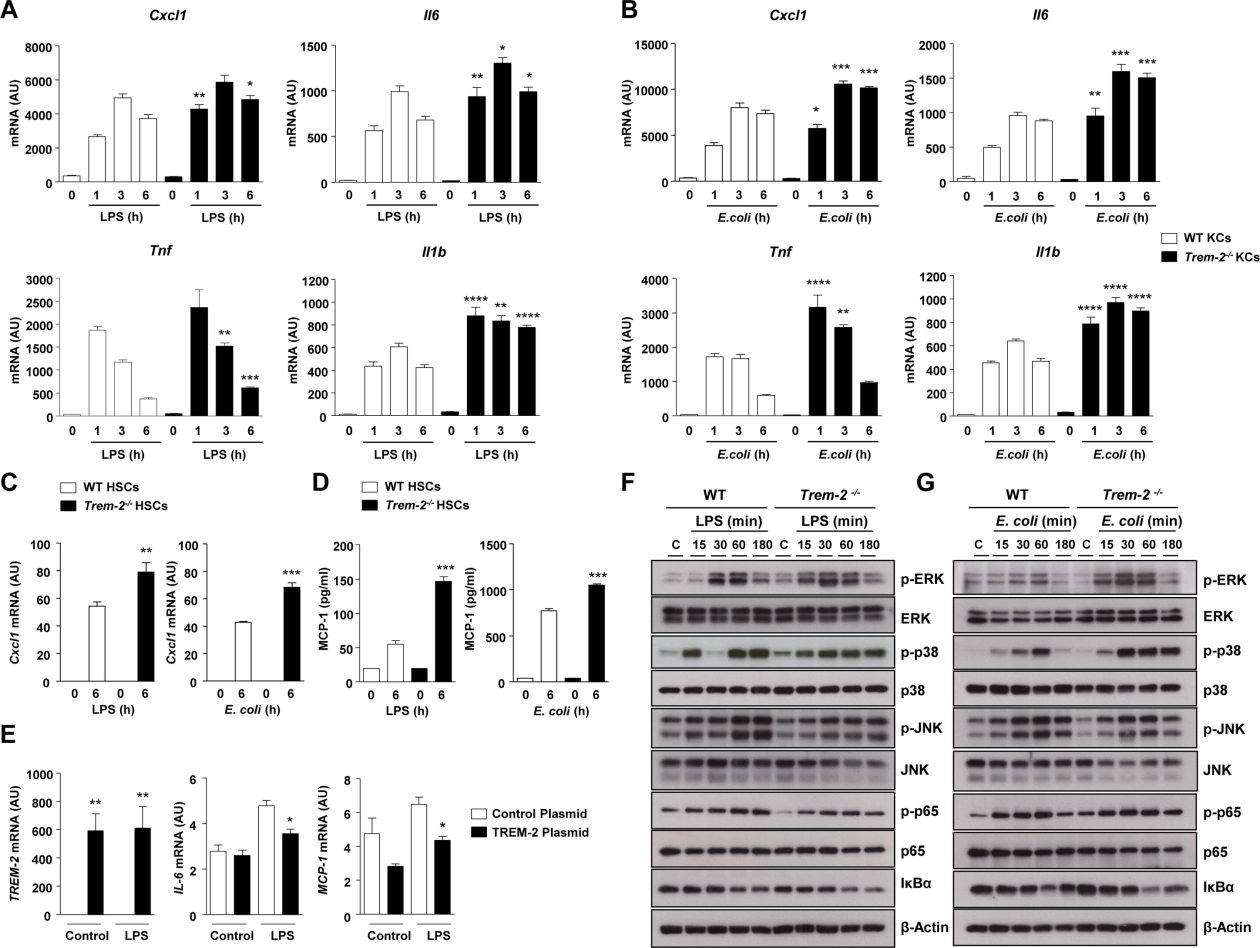
Cytokine responses and mitogen activated protein kinase (MAPK) signalling in WT and *Trem-2^-/-^* non-parenchymal hepatic cells after TLR4 stimulation. (A,B) WT and *Trem-2^-/-^* KCs were treated with LPS (100 ng/mL) (A) or (B) heat-killed *E. coli* (2×10^7^ CFU/mL) for the indicated time points (n=4–5 per condition and time point) and levels of *Cxcl1*, *Tnf*, *Il6* and *Il1b* were determined by qRT-PCR. (C, D) *Cxcl1* (C) and MCP-1 (D) levels in WT and *Trem-2^-/-^* activated mouse HSCs treated with 2×10^7^ CFU/mL heat-killed *E. coli* or 100 ng/mL LPS. n=3 (*E. coli*) and 4 (LPS). (E) Human HSC LX-2 cells were transfected with a control or *TREM-2* overexpressing plasmid (n=4 per condition) and 36 hours post-transfection stimulated with 100 ng/mL LPS for 3 hours and levels of *TREM-2*, *MCP-1* and *IL-6* determined by qRT-PCR. (F,G) WT and *Trem-2^-/-^* KCs were treated with 100 ng/mL LPS (F) or 2×10^7^ CFU/mL heat-killed *E. coli* (G) for the indicated time points and phosphorylation of ERK1/2, p38, JNK, p65 and IκB-α degradation was determined by western blotting. Data represent mean±SEM and *, **, ***, **** denote a P value of ˂0.05, ˂0.01, ˂0.001 and <0.0001, respectively versus WT at the same time point (one-way analysis of variance, followed by Tukey’s posthoc test). Data in (C,D) are representative of two independent experiments. *Cxcl1*, C-X-C motif chemokine ligand 1; ERK, extracellular regulated kinase; HSCs, hepatic stellate cells; JNK, Jun N-terminal kinase; KCs, Kupffer cells; LPS, lipopolysaccharide; MCP-1, monocyte chemoattractant protein-1; TREM, triggering receptor expressed on myeloid cells; WT, wild type.

**Figure 6:**
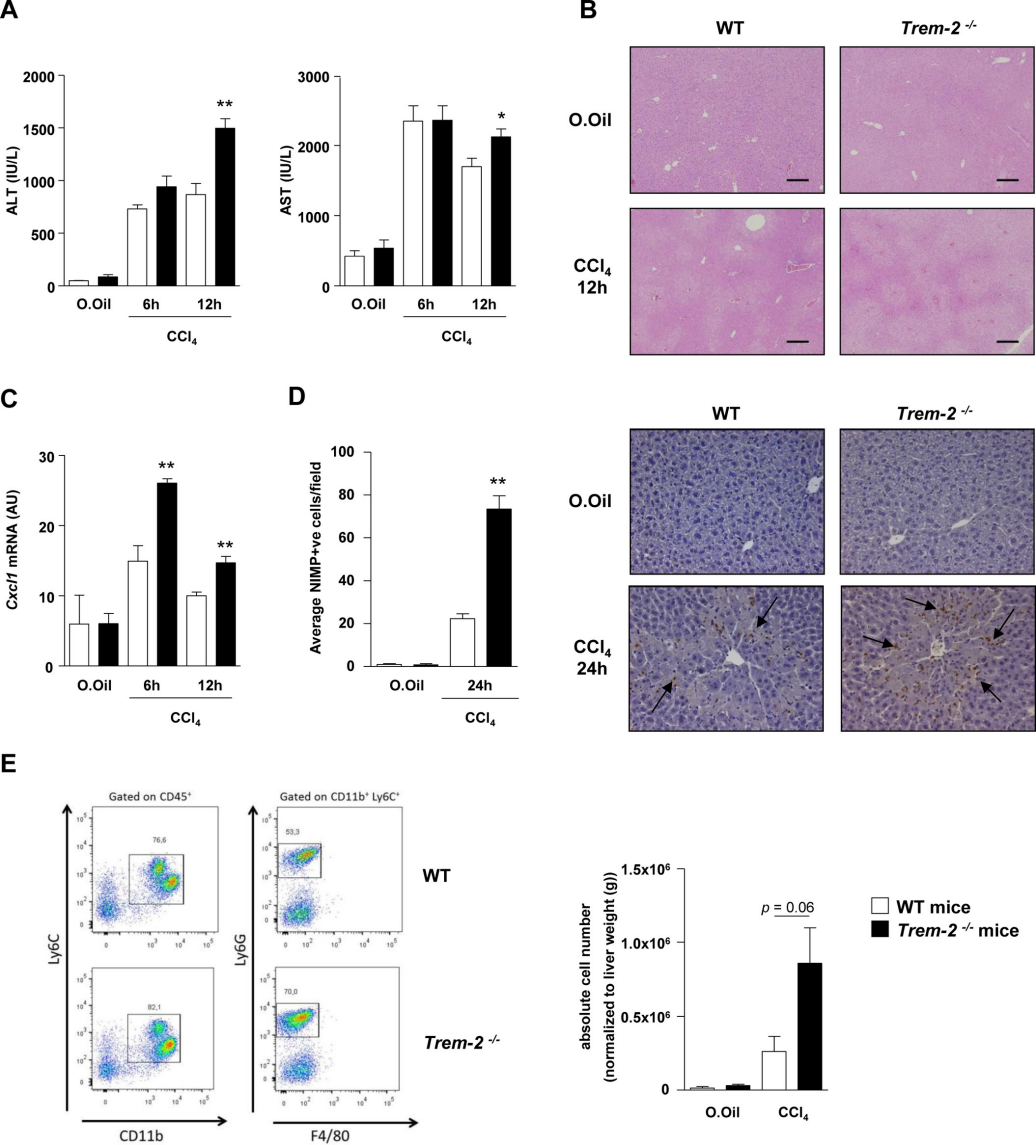
TREM-2 blunts acute CCl_4_ induced hepatic inflammation and injury. (A–C) Serum AST/ALT (A), representative H&E stains of liver (B) and qRT-PCR of liver *Cxcl1* (C) postacute CCl_4_ treatment of WT and *Trem-2^-/-^* mice for the indicated times. (D) Representative images of liver sections immunostained for neutrophils (anti-neutrophil marker (NIMP)) of WT and *Trem-2^-/-^* mice 24 hours after acute CCl_4_ treatment. Arrows denote positively stained cells. Manual counts for NIMP-positive cells in livers post-CCl_4_ treatment are depicted. (E) Representative flow cytometry plot of hepatic neutrophils of WT and *Trem-2^-/-^* mice 24 hours postacute CCl_4_ treatment. Total number of neutrophils normalised to liver weight are indicated. All data represent mean±SEM and *, ** denote a P value of ˂0.05 and ˂0.01, respectively versus WT at the same time point. n=5 mice per condition (A–C), three mice per condition (D) and n=6–10 mice per condition and are pooled data from two independent experiments (E). Statistical analysis used was unpaired Student’s t-test. Scale bar in (B) indicates 200 µm. ALT, alanine aminotransferase; AST, aspartate aminotransferase; CCl_4_, carbon tetrachloride; *Cxcl1*, C-X-C motif chemokine ligand 1; TREM, triggering receptor expressed on myeloid cells; WT, wild type.

### KC and HSC expressed TREM-2 dampens TLR4-mediated inflammation

We next focused on the effects of deletion of TREM-2 on the inflammatory properties of resident KCs and HSCs (figure 2) following TLR activation. While there was no effect of TREM-2 deficiency within KCs on LPS-driven *Mcp1* transcription (online supplementary figure 7), LPS stimulated TREM-2 deficient KCs displayed increased induction of *Il6*, *Tnf*, *Il1b* and C-X-C motif chemokine ligand 1 (*Cxcl1*), as early as 1 hour post-treatment (figure 5A). As dysbiosis of gut bacteria is important in CCl_4_-induced liver injury and inflammation,^22 23^ we next set to more closely mimic this scenario by treating KCs with the intestinal bacterium *Escherichia coli* (*E. coli*). *E. coli* treatment also resulted in increased proinflammatory cytokine transcription in *Trem-2* ^-/-^ KCs compared with WT (figure 5B). ELISA data verified that lack of TREM-2 enhanced the induction of inflammatory mediators following challenge with either LPS or *E. coli* (online supplementary figure 8). As we had observed increased *Mcp1* production in vivo following chronic injury (figures 3 and 4), but no effect of *Trem-2* expression within KCs on LPS-driven induced MCP-1 production (online supplementary figure 7), we speculated that HSCs might be a source for MCP-1 production in vivo. Indeed, examining TLR4-driven inflammation in both genotypes of activated HSCs revealed that LPS and *E. coli* treatment resulted in augmented *Cxcl1* and MCP-1 levels in activated *Trem-2^-/-^* HSC versus WT (figure 5C,D). Augmented inflammation in *Trem-2^-/-^* opposed to WT HSC was specific for TLR4-dependent signalling as LPS but not interleukin (IL)-1β treatment resulted in enhanced inflammation (online supplementary figure 9A). While IL-1β and TNF-α treatment of KCs was in general less proinflammatory compared with LPS, TREM-2 deficiency only led to augmented inflammation in response to LPS, further suggesting that this receptor does not impinge directly on TNF-α and IL-1β signalling cascades in non-parenchymal cells and ratifying specificity for TLR4-mediated signalling (online supplementary figure 9B). To corroborate that TREM-2 could dampen TLR4 responses within HSCs, we next performed gain of function experiments. TREM-2 overexpression dampened LPS-induced *MCP-1* and *IL-6* levels within human LX2 HSCs (figure 5E). We conclude that TREM-2 attenuates TLR4-mediated inflammatory responses of both KC and HSC.

**Figure 7:**
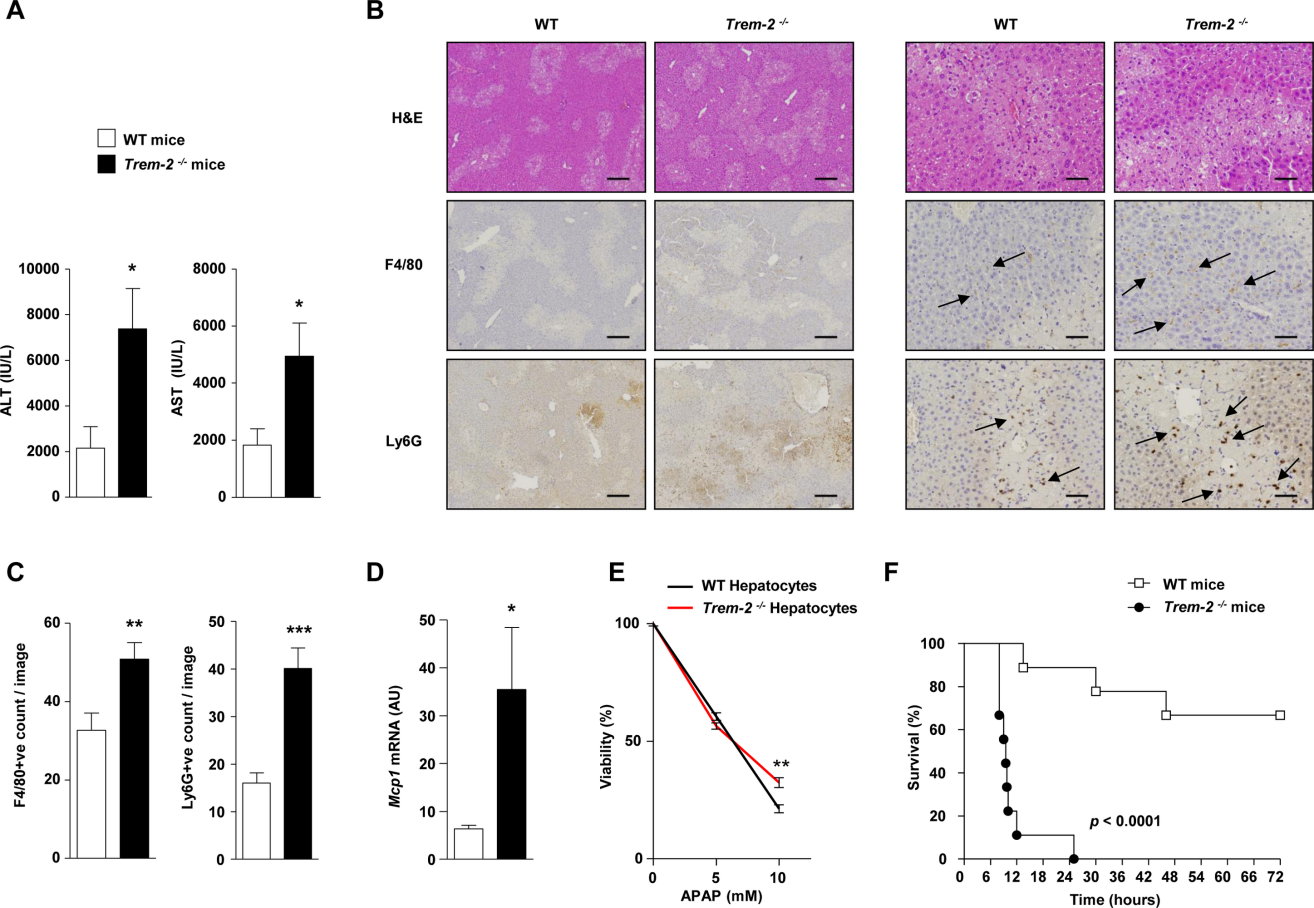
TREM-2 dampens acetaminophen-induced liver injury. (A) WT and *Trem-2^-/-^* mice were injected with 300 mg/kg APAP and 24 hours postinjury ALT and AST levels were determined. (B) Representative H&E, F4/80 and Ly-6G stains from the groups of mice are depicted. *Trem-2^-/-^* mouse liver exhibit more extensive parenchymal necrosis compared with WT liver. Arrows denote positively stained cells. (C) Manual counts for F4/80 and Ly6G positive cells in livers post-APAP treatment. (D) WT and *Trem-2^-/-^* mice were injected with 500 mg/kg APAP, and liver *Mcp1* levels were determined. (E) WT and *Trem-2^-/-^* hepatocytes were treated with indicated doses of APAP for 24 hours and cellular viability evaluated. (F) WT and *Trem-2^-/-^* mice were injected with 750 mg/kg APAP and survival was monitored. Data represent mean±SEM and *, **, ***, **** denote a P value of ˂0.05, ˂0.01, ˂0.001 and <0.0001, respectively versus WT. n=5 mice per genotype (A–D), 6 hepatocytes per genotype (E) or 9 mice per genotype (F). Statistical analysis used was unpaired Student’s t-test (A–E) and Log-rank (Mantel-Cox) test (F). Scale bar in (B) indicates 50 µm (left panel) or 200 µm (right panel). Data in (E) are representative of two independent experiments. ALT, alanine aminotransferase; AST, aspartate aminotransferase; APAP, acetaminophen; TREM, triggering receptor expressed on myeloid cells; WT, wild type.

**Figure 8:**
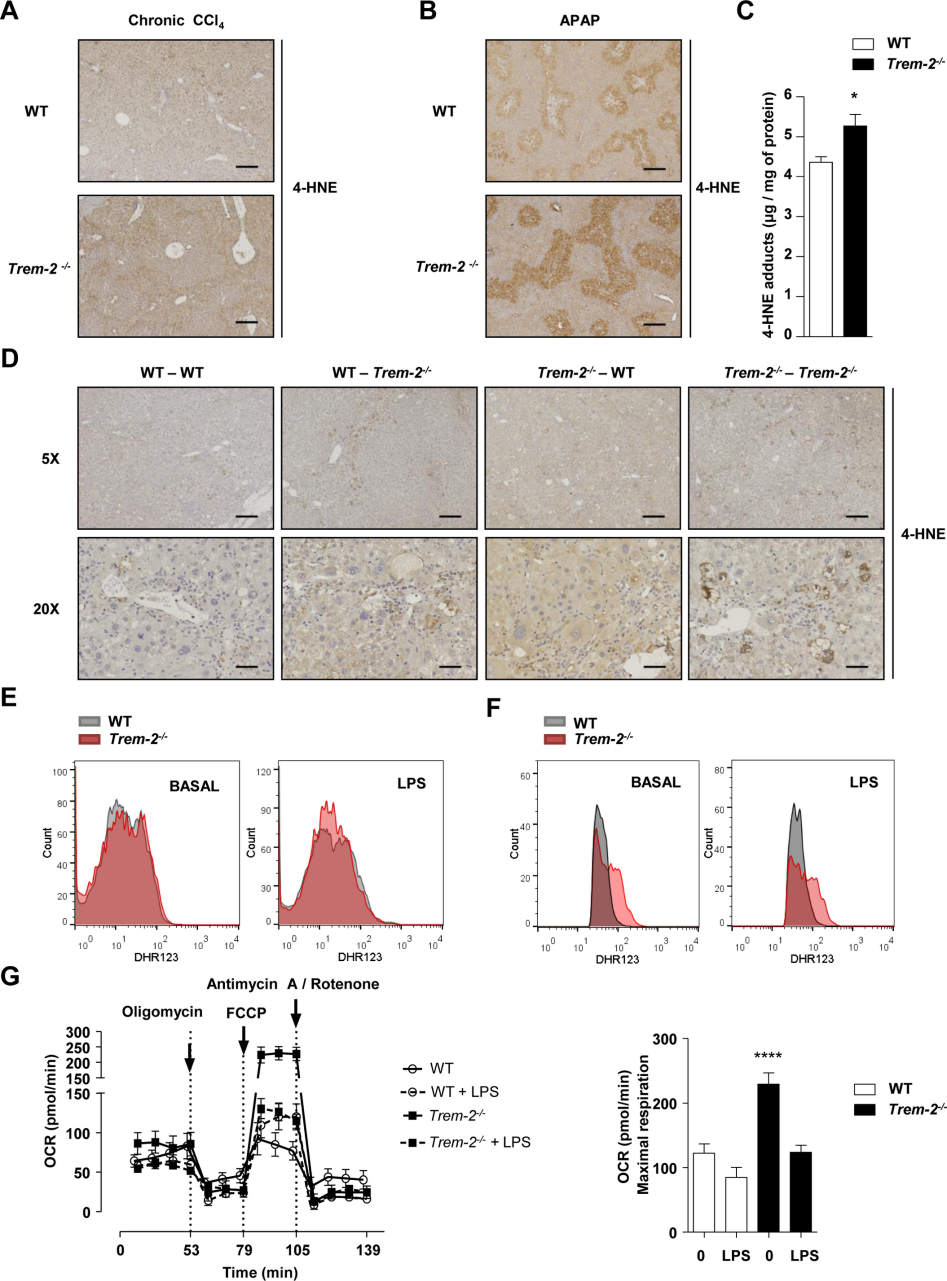
TREM-2 impacts hepatic lipid peroxidation and macrophage ROS levels. (A,B) Representative hepatic 4-HNE stain from WT and *Trem-2^-/-^* mice that were either treated with CCl_4_ for 8 weeks and sacrificed 1 day after the last CCl_4_ injection (A) or injected with 300 mg/kg APAP for 24 hours (B). n=3 mice per genotype (olive oil) or 4–8 mice per genotype (CCl_4_) or 5 mice per genotype (APAP). (C) 4-HNE content was determined by ELISA in livers of mice injected with 300 mg/kg APAP for 24 hours. n=5–7 per genotype. (D) Representative hepatic 4-HNE stain from WT mice reconstituted with WT-GFP^+^BM (WT-WT), *Trem-2^-/-^* mice reconstituted with *Trem-2^-/-^*-GFP^+^BM (*Trem-2^-/-^-Trem-2^-/-^*) or chimeric mice (WT-*Trem-2^-/-^*) and (*Trem-2^-/-^*-WT) that were treated with CCl_4_ for 8 weeks and sacrificed 1 day after the last CCl_4_ injection. n=3 per genotype (olive oil) and 3–5 per genotype (CCl_4_). Scale bar 5× indicates 50 µm and 20× indicates 200 µm. (E,F) WT and *Trem-2^-/-^* KCs (E) or BMDM (F) were treated with 100 ng/mL LPS for 3 hour and total cellular ROS levels were determined using flow cytometry for dihydrorhodamine 123. n=3–4 per genotype and condition and a representative histogram is shown. (G) Oxygen consumption rate of naive and LPS-treated BMDM was evaluated. n=4–5 per condition. Data in (C,G) represent mean ±SEM and * and **** denote a P value of< 0.05 and  <0.0001 versus WT. Data in (E) are representative of 3 and (F,G) of two independent experiments. Scale bar in (A,B) indicates 50 µm. APAP, acetaminophen; BMDM, bone marrow derived macrophages; CCl_4_, carbon tetrachloride; GFP, green fluorescent protein; 4-HNE, 4-hydroxynonenal; KCs, Kupffer cells; LPS, lipopolysaccharide; ROS, reactive oxygen species; TREM, triggering receptor expressed on myeloid cells; WT, wild type.

**Figure 9:**
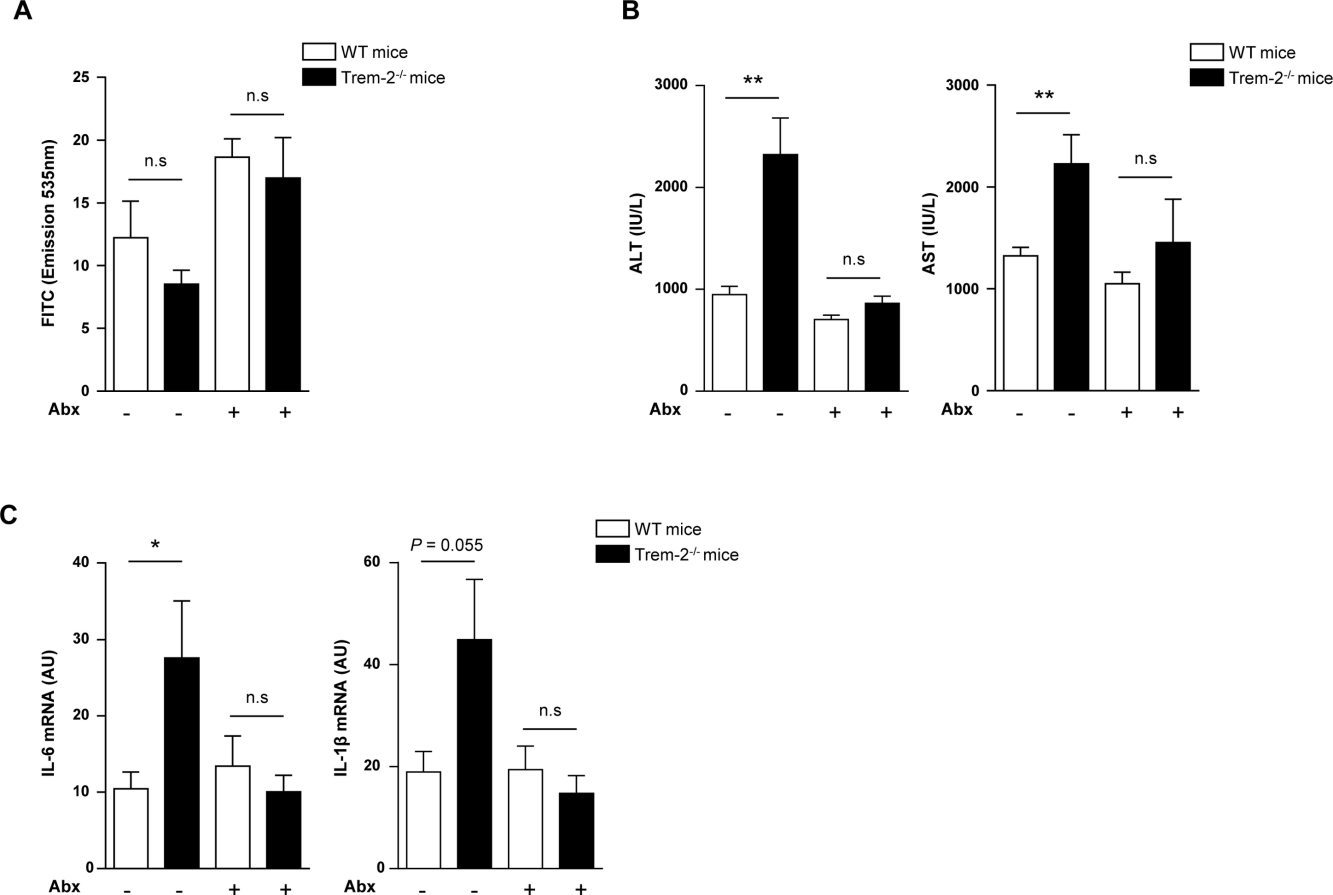
PAMPs are upstream of TREM-2 during liver injury (A–C) WT and *Trem-2^-/-^* mice that either received antibiotics or not in their drinking water for 4 weeks were injured acutely with CCl_4_ and 8 hours postinjection were orally gavaged with 4KDa-fluorescein isothiocyanate (FITC) Dextran and sacrificed 4 hours later. Serum levels of FITC Dextran (A), AST/ALT (B) and qRT-PCR of liver *Il6 and Il1b* (*C)* were determined. Data represent mean±SEM and *, ** denote a P value of ˂0.05 and ˂0.01, respectively versus the indicated genotype and condition (Student’s t-test). n=8–9 mice per genotype and condition. Abx, antibiotics; ALT, alanine aminotransferase; AST, aspartate aminotransferase; CCl_4_, carbon tetrachloride; IL, interleukin; PAMPs, pathogen-associated molecular patterns; TREM, triggering receptor expressed on myeloid cells; WT, wild type.

TREM-2 has been reported to modulate MAPK signalling and phosphorylation including ERK, p38 and Jun N-terminal kinase (JNK) phosphorylation, which are early and critical signal transduction events in TLR4-induced cytokine production.^11 24 25^ We next examined effects of TREM-2 on TLR4-mediated MAPK activation. *E. coli*-induced phosphorylation of ERK and p38 was potentiated in *Trem-2^-/-^* KCs, while there was a minor decrease in JNK activation, compared with WT KCs (figure 5G). Whereas decreases in JNK activation were more pronounced in response to LPS in *Trem-2^-/-^* compared with WT KCs, LPS-induced ERK phosphorylation was similarly augmented in *Trem2^-/-^* KC, suggesting TREM-2 functions to dampen the activation of ERK downstream of TLR4 (figure 5F,G). However, we observed no effect of TREM-2 deletion on TLR4-mediated nuclear factor kappa B (NF-κB) activation (figure 5F,G). We conclude that within resident liver macrophages, TREM-2 dampening of TLR4-driven inflammation is associated with an early attenuation of MAPK phosphorylation, particularly ERK activation.

### TREM-2 blunts acute CCl_4_ and APAP-induced liver inflammation and injury

Given the influence of TREM-2 on acute inflammatory responses of hepatic non-parenchymal cells, we determined if the receptor is functionally involved in controlling the response to acute toxic liver injury. We therefore performed two distinct acute liver injury models, administering mice with either a single dose of CCl_4_ or APAP overdose, the latter being a common cause of acute human liver failure associated with mitochondrial dysfunction, stress, lipid peroxidation and liver necrosis.^26^

Twelve hours postacute CCl_4_ injury, *Trem-2^-/-^* mice displayed elevated liver transaminases compared with WT, this effect was associated with elevated levels of the neutrophil chemoattractant *Cxcl1* and increased hepatic neutrophil recruitment 24 hours postinjury, as determined using immunohistochemistry (figure 6A–D). By contrast, there were unremarkable effects of TREM-2 deletion on macrophage recruitment (data not shown). To confirm these data, we next isolated inflammatory cells from the livers of both genotypes of mice 24 hours post CCl_4_ injury and using flow cytometry again observed a strong tendency (P=0.06) for elevated hepatic neutrophil numbers in *Trem2^-/-^* mice compared with WT controls (figure 6E).

Similar to acute CCl_4_ injury, following sublethal APAP intoxication, *Trem-2^-/-^* mice displayed augmented transaminase levels, more extensive parenchymal necrosis and increased neutrophil content compared with WT (figure 7A–C). However, in this model, *Trem-2^-/-^* mice also displayed elevated macrophage content, which was associated with elevated levels of *Mcp1* (figure 7B–D). These data suggested that enhanced pathogen-associated molecular pattern (PAMP)-driven inflammation from non-parenchymal liver cells in *Trem-2^-/-^* mice might be a driver for the elevated liver damage. To exclude a toxic effect of APAP on parenchymal cells, we isolated both genotypes of hepatocytes, treated them with APAP and evaluated cell viability. While APAP dose dependently killed hepatocytes, cell death was not elevated in TREM-2 deficient hepatocytes, in fact, surprisingly there was a small although statistically significant tendency that viability was increased (figure 7E). To further examine the consequences of the enhanced liver damage in *Trem-2^-/-^* mice, we administered both genotypes of mice with a lethal dose of APAP and monitored survival. Strikingly, while 70% of WT mice survived up until 72 hours, all *Trem-2^-/-^* mice died within 25 hours (figure 7F). We conclude that TREM-2 is critical for appropriate dampening of the acute liver damage response and its associated inflammatory reaction, with the immune receptor therefore being essential for preventing lethal APAP intoxication.

### TREM-2 suppresses injury-induced hepatic ROS and lipid peroxidation

Previous observations from our laboratory indicate that TREM-2 can modulate peroxisome proliferator-activated receptor (PPAR) and nuclear receptor activities that regulate lipid-associated pathways.^27^ Inflammation, particularly that mediated by CCl_4_ and APAP intoxication is associated with enhanced lipid peroxidation, which can result in hepatocyte death.^28–30^ In search for a unified mechanism behind the elevated liver injury of *Trem-2^-/-^* mice following chronic CCl_4_ and acute APAP intoxication, we next monitored levels of 4-hydroxynonenal (4-HNE), a surrogate for lipid peroxidation.^30^ Both, at peak injury following chronic CCl_4_ treatment and APAP intoxication, elevated 4-HNE levels were observed in the livers of *Trem-2^-/-^* mice (figure 8A,B). To confirm these data, in an independent experiment, we next isolated livers from both genotypes of mice 24 hours post APAP treatment and could indeed observe significantly elevated 4-HNE levels as determined using ELISA (figure 8C). Examining 4-HNE staining revealed that lipid peroxidation appeared to be most intense around hepatic venules, where inflammatory cells reside, suggesting that elevated lipid peroxidation might occur through enhanced reactive oxygen species (ROS) levels within resident or infiltrating immune cells. Supporting this idea, elevated lipid peroxidation following chronic CCl_4_ treatment was only observed when TREM-2 was deficient within resident and infiltrating immune cells (figure 8D). As the ability of TREM-2 to dampen liver damage following chronic CCl_4_ treatment depended on TREM-2 expression in both resident and infiltrating cells (figure 4), these data further linked elevated lipid peroxidation to injury and additionally suggested that this effect could be associated with enhanced ROS levels within *Trem-2^-/-^* resident and liver infiltrating immune cells. However, examining ROS levels within both genotypes of KCs revealed similar levels in both basal and LPS-driven ROS (figure 8E). Considering increased lipid peroxidation in *Trem-2^-/-^* mice was associated with elevated MCP-1 and newly recruited monocyte derived hepatic macrophage levels, we next asked if elevated liver damage in *Trem-2^-/-^* mice could be associated with differential ROS levels within injury-associated inflammatory monocytes that repopulate the liver. We used BMDM to model this. Strikingly, *Trem-2^-/-^* BMDM exhibited higher baseline and LPS-induced ROS levels (figure 8F). Mitochondria via the process of oxidative phosphorylation and the NADPH oxidase complex produce ROS, so we next set out to validate these findings as well as determine the cellular source for the elevated ROS in *Trem-2^-/-^* macrophages. Intriguingly, when assessing mitochondrial activity, we observed that *Trem-2^-/-^* BMDM exhibited higher baseline oxidative phosphorylation and maximal respiration, suggesting that this metabolic signature might be responsible for the enhanced ROS (figure 8G). These data suggest that TREM-2 affects lipid peroxidation and subsequent damage of hepatocytes by skewing metabolic pathways and ROS generation within newly recruited liver macrophages following liver injury.

### PAMPs are upstream of TREM-2 during liver injury

Thus far our observations indicated an importance for TREM-2 in dampening hepatic injury and inflammation. In an effort to demonstrate that the upstream driver for acute liver injury in TREM-2 deficient mice was PAMPs and associated TLR4 signalling downstream of gut bacteria, we administered both genotypes of mice antibiotics and evaluated liver injury and inflammation post-CCl_4_ treatment versus animals that did not receive antibiotics. Eight hours postacute CCl_4_ injection, we orally gavaged all groups of mice with 4 kDa FITC-Dextran, to evaluate intestinal permeability as previously described^22^ and sacrificed all animals 4 hours later, that is, we administered CCl_4_ for a total of 12 hours. Although there was a trend that antibiotic treatment increased intestinal permeability, serum FITC levels were unaltered between genotypes in either the presence or absence of antibiotics post-CCl_4_ treatment (figure 9A). *Trem-2****^-/-^*** mice exhibited exaggerated transaminase release compared with WT following acute CCl_4_ treatment, as previously observed (figure 7A) and notably antibiotics abolished this (figure 9B). Evaluating transcript levels of *Il1b* and *Il6*, revealed that antibiotic treatment also reverted the augmented hepatic inflammation of *Trem-2****^-/-^*** mice to WT levels (figure 9C). Together, these important data show that following acute liver injury, differences in altered intestinal permeability of TREM-2 deficient mice do not contribute to their exaggerated liver damage. They further support the concept that TREM-2 dampens PAMP-derived signals that emanate from gut bacteria which perpetuate hepatic injury and inflammation.

## Discussion

Unresolved inflammation is a pathogenic component of liver disease and contributes to progressive liver damage and fibrosis. Altered intestinal permeability during liver injury results in leakage of PAMPs that are recognised by non-parenchymal liver cells and injury recruited cells. These cells release a variety of proinflammatory mediators including cytokines, chemokines, lipids and ROS that contribute to the demise of hepatocytes. Illustrating the importance of innate immunity, mice deficient in key mediators of LPS signalling such as TLR4, CD14 and MyD88 display better outcomes during liver disease.^4 31 32^ Here, we tested a hypothesised anti-inflammatory role for TREM-2 in liver disease.

We show that TREM-2 expression dramatically rises during various forms of liver injury in both mice and humans and that hepatic mouse TREM-2 is expressed on non-parenchymal KCs and HSCs. Nonetheless, we cannot be certain that in other species, including humans, TREM-2 is not expressed in hepatocytes during liver injury. Notably, although TREM-2 receptor expression has previously been detected on a variety of resident macrophages, including KCs,^8 10 13 27^ to our knowledge, this is the first study demonstrating TREM-2 expression on quiescent and activated HSCs, expanding on studies showing TREM-1 expression on this important immune and fibrogenic cell type.^12^ Further, we disclose that during HSC activation, TREM-2 is dramatically increased in a conserved manner and is upregulated within HSCs during chronic liver injury. However, TREM-2 exerted unremarkable effects on HSC activation in vitro and in the context of chronic liver injury, effects consistent with minor effects of TREM-2 on fibrosis and the critical fibrogenic mediator, TGFβ−1 during chronic injury.^20 21^ Further, as the net amount of collagen deposition is controlled by matrix metalloproteinases and their inhibitors, augmented *Mmp13* levels observed in *Trem-2^-^* ^/-^ mice during repetitive injury could play a role in fibrosis resolution.^33^ Interestingly, although HSCs are not professional phagocytes, phagocytosis of apoptotic bodies by HSCs has been documented and postulated to promote HSC activation, survival, TGFβ−1 and collagen production.^34–36^ In some cell types, TREM-2 has been described to be important for the clearance of bacteria and apoptotic cells.^11 16 27 37^ Thus, defects in the uptake of apoptotic bodies by *Trem-2^-^* ^/-^ HSCs could additionally contribute to the minor effects of TREM-2 on fibrosis observed during chronic CCl_4_ injury. To summarise, our data indicate that TREM-2 primarily limits hepatocellular injury, a process that appears to be linked to its negative regulatory role during inflammation.

Similar to BM macrophages,^8^ TREM-2 dampened TLR4-driven inflammation in KCs and HSCs. Higher inflammation in *Trem-2^-^* ^/-^ KCs correlated with effects of TREM-2 on TLR4-induced ERK activation, consistent with studies demonstrating effects of TREM-2 on the ERK but not the NF-κB pathway.^11 24^ During acute CCl_4_-mediated injury, *Trem-2^-/-^* mice exhibited enhanced numbers of liver neutrophils compared with WT, which correlated with elevated hepatic levels of the neutrophil chemoattractant CXCL1 and liver damage.^38^ The ability of TREM-2 to dampen CXCL1 and neutrophil recruitment following acute liver injury is consistent with how pulmonary expressed TREM-2 reacts during early pneumococcal infection.^27^ Similarly, following acute APAP intoxication, augmented neutrophil influx was observed in *Trem-2^-/-^* livers. However, unlike acute CCl_4_ treatment hepatic macrophage content was also elevated in *Trem-2^-/-^* livers. Although the relative contribution of macrophages versus neutrophils to the enhanced liver injury of *Trem-2^-/-^* mice in the various models of liver injury remains to be determined, we importantly show that (1) TREM-2 dampens liver damage in all models tested; (2) lack of TREM-2 is associated with worsened survival outcome following APAP challenge; (3) gut-derived PAMPs are a driver for acute liver injury of TREM-2 deficient mice.

In terms of mechanistic parallels between APAP intoxication and chronic CCl_4_ injury, we observed significantly enhanced *Mcp1* levels in *Trem-2^-^* ^/-^ versus WT livers in both models, which was associated with more damage, macrophage levels and lipid peroxidation. Although resident KCs have a prenatal origin and are generated from the yolk sac and/or fetal liver, during liver injury, circulating Ly6C^+^ monocytes are recruited to the injured liver in a MCP-1/CCR-2 dependent manner, where they can contribute to injury.^39–41^ Indeed, it has recently been shown that hepatic infiltrating monocyte derived macrophages exert proinflammatory functions early after APAP and thereby promote liver injury. Consequently, reducing monocyte infiltration via MCP-1/CCR2 inhibition attenuates APAP-induced liver injury.^42^ Together, these data indicate that during acute toxic hepatic injury, the recruitment of monocytes to the liver perpetuates parenchymal damage, a hypothesis that is supported by studies indicating that KCs play a protective role in APAP-induced injury.^43^ We now add to this knowledge by highlighting the importance of TREM-2 signalling for suppressing recruitment of extrahepatic macrophages in toxic liver injury.

Our data suggest that TREM-2 dampens TLR4-dependent MCP-1 production within HSCs, impacting hepatic macrophage recruitment. Furthermore, TREM-2 expressed on these infiltrating macrophages blunts ROS production, thus limiting ROS-mediated hepatocellular lipid peroxidation and cell death. Effects of TREM-2 on ROS within infiltrating macrophages alone appears insufficient to dampen liver damage, as WT mice reconstituted with *Trem-2^-/-^* BM do not display more liver damage than WT mice with WT BM. Rather, our data suggest that TREM-2 effects on TLR4-dependent inflammatory events in HSCs is critical for setting the stage for hepatic macrophage recruitment, a process influenced by HSC-derived MCP-1 and by ROS and cytokine production from infiltrating immune cells. These observations are concordant with data demonstrating that liver steatosis sensitises to CCl_4_ hepatotoxicity, in part through oxidative stress.^44^ Supporting a cross-talk between infiltrating monocytes and HSCs in liver injury, purified Cd11b^+^F4/80^+^Ly6C^+^ cells derived from CCl_4_ treated mice can directly activate HSCs, promoting hepatocellular damage.^45^ In this regard, it is interesting that infiltrating inflammatory Ly6C^+^ macrophages downregulate Ly6C on uptake of apoptotic hepatocytes and this is associated with a tissue protective phenotype following alcoholic-induced liver injury.^46^ Although the exact nature of infiltrating macrophages during injury in *Trem-2^-^*^/-^ mice in the models of liver injury requires further examination, our data, along with published observations, suggest they could be important for TREM-2 to limit hepatocellular damage following APAP intoxication and chronic CCl_4_ injury. Hepatocyte death would presumably release a variety of danger-associated molecular patterns, including heat-shock proteins, purine metabolites and mitochondrial DNA that can further provoke a vicious cycle of inflammation, macrophage recruitment and cell death.^28^ Such a hepatotoxic feed forward loop could be present and elevated in *Trem-2^-^*^/-^ livers during chronic injury and APAP intoxication. Further, effects on TREM-2 on liver lipid metabolism are reminiscent with very recent studies indicating TREM-2 alters brain lipid metabolism and purogenic signalling in the context of cuprizone-induced oligodendrocyte injury and that TREM-2 can sense lipids.^17 47^ Highlighting the importance of TREM-2 in lipid metabolism, recent data indicate that TREM-2 promotes adipogenesis and in vivo high fat diet fed mice overexpressing TREM-2 were more obese and developed elevated hepatic steatosis versus controls.^48^ Although the authors of the aforementioned study did not perform an extensive analysis of TREM-2’s function in hepatic injury in this context, these data suggest TREM-2’s role in hepatocellular injury during non-alcoholic fatty liver disease may be distinct and complex, involving not just its immune but also adipogenic and lipid regulatory functions.^47 48^

This is the first study demonstrating a role for TREM-2 in toxic liver injury and is reminiscent of published effects of TREM-2 in injury responses in other organ and tissue systems. For example, in the colon, TREM-2 has been reported to be important in wound-healing.^15^ In the brain, TREM-2 functions to clear apoptotic neurons and limit microglial proinflammatory responses.^11 16^ Our work suggests that TREM-2 could be an attractive target for promoting the resolution of inflammation during liver injury and for the prevention of parenchymal cell death, this akin to events described for TREM-2 in neuroinflammatory diseases and sepsis.^16 49^

## References

[1] Thobe BM, Frink M, Hildebrand F, et al The role of MAPK in Kupffer cell toll-like receptor (TLR) 2-, TLR4-, and TLR9-mediated signaling following trauma-hemorrhage. J Cell Physiol 2007;210:667–75. 10.1002/jcp.20860 17117477

[2] Paik YH, Schwabe RF, Bataller R, et al Toll-like receptor 4 mediates inflammatory signaling by bacterial lipopolysaccharide in human hepatic stellate cells. Hepatology 2003;37:1043–55. 10.1053/jhep.2003.50182 12717385

[3] Liu S, Gallo DJ, Green AM, et al Role of toll-like receptors in changes in gene expression and NF-kappa B activation in mouse hepatocytes stimulated with lipopolysaccharide. Infect Immun 2002;70:3433–42. 10.1128/IAI.70.7.3433-3442.2002 12065483 PMC128073

[4] Seki E, De Minicis S, Osterreicher CH, et al TLR4 enhances TGF-beta signaling and hepatic fibrosis. Nat Med 2007;13:1324–32. 10.1038/nm1663 17952090

[5] Shen XD, Ke B, Zhai Y, et al Absence of toll-like receptor 4 (TLR4) signaling in the donor organ reduces ischemia and reperfusion injury in a murine liver transplantation model. Liver Transpl 2007;13:1435–43. 10.1002/lt.21251 17902130

[6] Wang Y, Hu Y, Chao C, et al Role of IRAK-M in alcohol induced liver injury. PLoS One 2013;8:e57085 10.1371/journal.pone.0057085 23437317 PMC3578822

[7] Ramsey HE, Da Silva CG, Longo CR, et al A20 protects mice from lethal liver ischemia/reperfusion injury by increasing peroxisome proliferator-activated receptor-alpha expression. Liver Transpl 2009;15:1613–21. 10.1002/lt.21879 19877201 PMC2976064

[8] Turnbull IR, Gilfillan S, Cella M, et al Cutting edge: TREM-2 attenuates macrophage activation. J Immunol 2006;177:3520–4. 10.4049/jimmunol.177.6.3520 16951310

[9] Bouchon A, Dietrich J, Colonna M Cutting edge: inflammatory responses can be triggered by TREM-1, a novel receptor expressed on neutrophils and monocytes. J Immunol 2000;164:4991–5. 10.4049/jimmunol.164.10.4991 10799849

[10] Sharif O, Knapp S From expression to signaling: roles of TREM-1 and TREM-2 in innate immunity and bacterial infection. Immunobiology 2008;213:701–13. 10.1016/j.imbio.2008.07.008 18926286

[11] Takahashi K, Rochford CD, Neumann H Clearance of apoptotic neurons without inflammation by microglial triggering receptor expressed on myeloid cells-2. J Exp Med 2005;201:647–57. 10.1084/jem.20041611 15728241 PMC2213053

[12] Liao R, Sun TW, Yi Y, et al Expression of TREM-1 in hepatic stellate cells and prognostic value in hepatitis B-related hepatocellular carcinoma. Cancer Sci 2012;103:984–92. 10.1111/j.1349-7006.2012.02273.x 22417086 PMC7685080

[13] Gonçalves LA, Rodrigues-Duarte L, Rodo J, et al TREM2 governs Kupffer cell activation and explains belr1 genetic resistance to malaria liver stage infection. Proc Natl Acad Sci U S A 2013;110:19531–6. 10.1073/pnas.1306873110 24218563 PMC3845107

[14] Chen LC, Laskin JD, Gordon MK, et al Regulation of TREM expression in hepatic macrophages and endothelial cells during acute endotoxemia. Exp Mol Pathol 2008;84:145–55. 10.1016/j.yexmp.2007.11.004 18222421 PMC2752215

[15] Seno H, Miyoshi H, Brown SL, et al Efficient colonic mucosal wound repair requires Trem2 signaling. Proc Natl Acad Sci U S A 2009;106:256–61. 10.1073/pnas.0803343106 19109436 PMC2629230

[16] Takahashi K, Prinz M, Stagi M, et al TREM2-transduced myeloid precursors mediate nervous tissue debris clearance and facilitate recovery in an animal model of multiple sclerosis. PLoS Med 2007:e124:4 (Epub ahead of print 12 Apr 2017). 17425404 10.1371/journal.pmed.0040124 PMC1851623

[17] Cantoni C, Bollman B, Licastro D, et al TREM2 regulates microglial cell activation in response to demyelination in vivo. Acta Neuropathol 2015;129:429–47. 10.1007/s00401-015-1388-1 25631124 PMC4667728

[18] Perugorria MJ, Murphy LB, Fullard N, et al Tumor progression locus 2/Cot is required for activation of extracellular regulated kinase in liver injury and toll-like receptor-induced TIMP-1 gene transcription in hepatic stellate cells in mice. Hepatology 2013;57:1238–49. 10.1002/hep.26108 23080298

[19] Mann J, Chu DC, Maxwell A, et al MeCP2 controls an epigenetic pathway that promotes myofibroblast transdifferentiation and fibrosis. Gastroenterology 2010;138:705–14. 10.1053/j.gastro.2009.10.002 19843474 PMC2819585

[20] Friedman SL Hepatic stellate cells: protean, multifunctional, and enigmatic cells of the liver. Physiol Rev 2008;88:125–72. 10.1152/physrev.00013.2007 18195085 PMC2888531

[21] Friedman SL Evolving challenges in hepatic fibrosis. Nat Rev Gastroenterol Hepatol 2010;7:425–36. 10.1038/nrgastro.2010.97 20585339

[22] Fouts DE, Torralba M, Nelson KE, et al Bacterial translocation and changes in the intestinal microbiome in mouse models of liver disease. J Hepatol 2012;56:1283–92. 10.1016/j.jhep.2012.01.019 22326468 PMC3357486

[23] Gómez-Hurtado I, Santacruz A, Peiró G, et al Gut microbiota dysbiosis is associated with inflammation and bacterial translocation in mice with CCl4-induced fibrosis. PLoS One 2011;6:e23037 10.1371/journal.pone.0023037 21829583 PMC3146520

[24] Bouchon A, Hernández-Munain C, Cella M, et al A DAP12-mediated pathway regulates expression of CC chemokine receptor 7 and maturation of human dendritic cells. J Exp Med 2001;194:1111–22. 10.1084/jem.194.8.1111 11602640 PMC2193511

[25] Guha M, Mackman N LPS induction of gene expression in human monocytes. Cell Signal 2001;13:85–94. 10.1016/S0898-6568(00)00149-2 11257452

[26] McGill MR, Williams CD, Xie Y, et al Acetaminophen-induced liver injury in rats and mice: comparison of protein adducts, mitochondrial dysfunction, and oxidative stress in the mechanism of toxicity. Toxicol Appl Pharmacol 2012;264:387–94. 10.1016/j.taap.2012.08.015 22980195 PMC3478469

[27] Sharif O, Gawish R, Warszawska JM, et al The triggering receptor expressed on myeloid cells 2 inhibits complement component 1q effector mechanisms and exerts detrimental effects during pneumococcal pneumonia. PLoS Pathog 2014;10:e1004167 10.1371/journal.ppat.1004167 24945405 PMC4055749

[28] Brenner C, Galluzzi L, Kepp O, et al Decoding cell death signals in liver inflammation. J Hepatol 2013;59:583–94. 10.1016/j.jhep.2013.03.033 23567086

[29] Manibusan MK, Odin M, Eastmond DA Postulated carbon tetrachloride mode of action: a review. J Environ Sci Health C Environ Carcinog Ecotoxicol Rev 2007;25:185–209. 10.1080/10590500701569398 17763046

[30] Das M, Boerma M, Goree JR, et al Pathological changes in pulmonary circulation in carbon tetrachloride (CCl4)-induced cirrhotic mice. PLoS One 2014;9:e96043 10.1371/journal.pone.0096043 24763616 PMC3999097

[31] Li Z, Yang S, Lin H, et al Probiotics and antibodies to TNF inhibit inflammatory activity and improve nonalcoholic fatty liver disease. Hepatology 2003;37:343–50. 10.1053/jhep.2003.50048 12540784

[32] Rivera CA, Adegboyega P, van Rooijen N, et al Toll-like receptor-4 signaling and Kupffer cells play pivotal roles in the pathogenesis of non-alcoholic steatohepatitis. J Hepatol 2007;47:571–9. 10.1016/j.jhep.2007.04.019 17644211 PMC2094119

[33] Higashiyama R, Inagaki Y, Hong YY, et al Bone marrow-derived cells express matrix metalloproteinases and contribute to regression of liver fibrosis in mice. Hepatology 2007;45:213–22. 10.1002/hep.21477 17187438

[34] Zhan SS, Jiang JX, Wu J, et al Phagocytosis of apoptotic bodies by hepatic stellate cells induces NADPH oxidase and is associated with liver fibrosis in vivo. Hepatology 2006;43:435–43. 10.1002/hep.21093 16496318

[35] Jiang JX, Mikami K, Venugopal S, et al Apoptotic body engulfment by hepatic stellate cells promotes their survival by the JAK/STAT and Akt/NF-kappaB-dependent pathways. J Hepatol 2009;51:139–48. 10.1016/j.jhep.2009.03.024 19457567 PMC2765371

[36] Canbay A, Taimr P, Torok N, et al Apoptotic body engulfment by a human stellate cell line is profibrogenic. Lab Invest 2003;83:655–63. 10.1097/01.LAB.0000069036.63405.5C 12746475

[37] N’Diaye EN, Branda CS, Branda SS, et al TREM-2 (triggering receptor expressed on myeloid cells 2) is a phagocytic receptor for bacteria. J Cell Biol 2009;184 215–23. 10.1083/jcb.200808080 19171755 PMC2654305

[38] Simpson KJ, Henderson NC, Bone-Larson CL, et al Chemokines in the pathogenesis of liver disease: so many players with poorly defined roles. Clin Sci 2003;104:47–63. 10.1042/cs1040047 12519087

[39] Schulz C, Gomez Perdiguero E, Chorro L, et al A lineage of myeloid cells independent of Myb and hematopoietic stem cells. Science 2012;336:86–90. 10.1126/science.1219179 22442384

[40] Zimmermann HW, Trautwein C, Tacke F Functional role of monocytes and macrophages for the inflammatory response in acute liver injury. Front Physiol 2012;3:56 10.3389/fphys.2012.00056 23091461 PMC3475871

[41] Zigmond E, Samia-Grinberg S, Pasmanik-Chor M, et al Infiltrating monocyte-derived macrophages and resident kupffer cells display different ontogeny and functions in acute liver injury. J Immunol 2014;193:344–53. 10.4049/jimmunol.1400574 24890723

[42] Mossanen JC, Krenkel O, Ergen C, et al Chemokine (C-C motif) receptor 2-positive monocytes aggravate the early phase of acetaminophen-induced acute liver injury. Hepatology 2016;64:1667–82. 10.1002/hep.28682 27302828

[43] Ju C, Reilly TP, Bourdi M, et al Protective role of Kupffer cells in acetaminophen-induced hepatic injury in mice. Chem Res Toxicol 2002;15:1504–13. 10.1021/tx0255976 12482232

[44] Donthamsetty S, Bhave VS, Mitra MS, et al Nonalcoholic fatty liver sensitizes rats to carbon tetrachloride hepatotoxicity. Hepatology 2007;45:391–403. 10.1002/hep.21530 17256749

[45] Karlmark KR, Weiskirchen R, Zimmermann HW, et al Hepatic recruitment of the inflammatory Gr1+ monocyte subset upon liver injury promotes hepatic fibrosis. Hepatology 2009;50:261–74. 10.1002/hep.22950 19554540

[46] Wang M, You Q, Lor K, et al Chronic alcohol ingestion modulates hepatic macrophage populations and functions in mice. J Leukoc Biol 2014;96:657–65. 10.1189/jlb.6A0114-004RR 25030420 PMC4163632

[47] Wang Y, Cella M, Mallinson K, et al TREM2 lipid sensing sustains the microglial response in an Alzheimer’s disease model. Cell 2015;160:1061–71. 10.1016/j.cell.2015.01.049 25728668 PMC4477963

[48] Park M, Yi JW, Kim EM, et al Triggering receptor expressed on myeloid cells 2 (TREM2) promotes adipogenesis and diet-induced obesity. Diabetes 2015;64:117–27. 10.2337/db13-1869 25114293

[49] Chen Q, Zhang K, Jin Y, et al Triggering receptor expressed on myeloid cells-2 protects against polymicrobial sepsis by enhancing bacterial clearance. Am J Respir Crit Care Med 2013;188:201–12. 10.1164/rccm.201211-1967OC 23721075

