# Supplementary material for: Non-parenchymal TREM-2 protects the liver from immune-mediated hepatocellular damage
Source: Gut. 2018 Jan 27;68(3):533–46. doi: 10.1136/gutjnl-2017-314107 (PMC6580759; doi:10.1136/gutjnl-2017-314107)
Supplement: Supplementary file 1 [file gutjnl-2017-314107supp001.pdf]

A

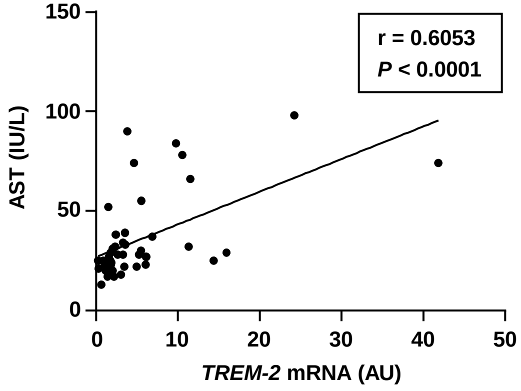

B

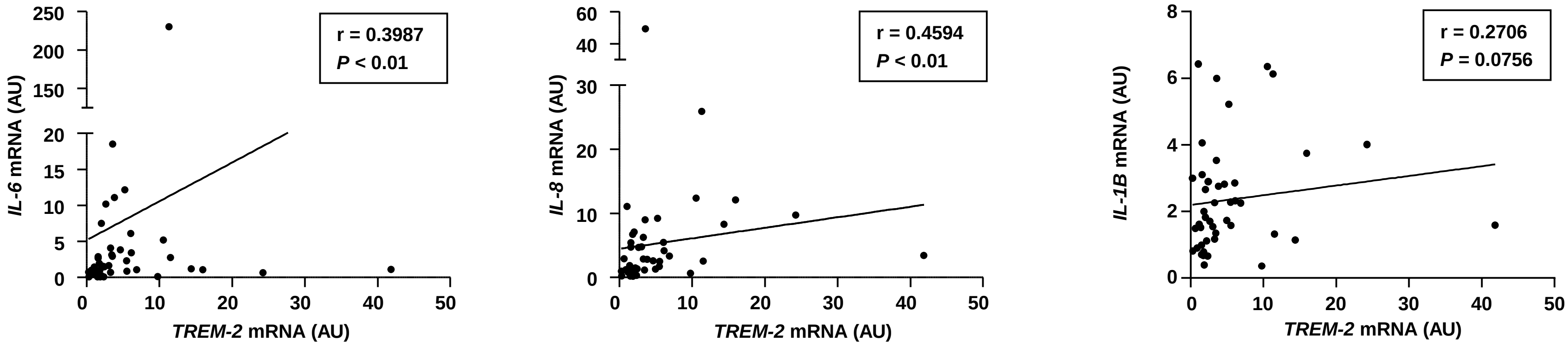

Supplementary Figure 1: Correlation of *TREM-2* levels with AST and pro-inflammatory cytokines.
qRT-PCR analysis of *TREM-2* in control human liver and cirrhotic samples was performed and correlated with (**A**) AST and (**B**) pro-inflammatory cytokine expression (*IL-6*, *IL-8* and *IL-1B*). n = 21 control cases and 23 cirrhotic livers.

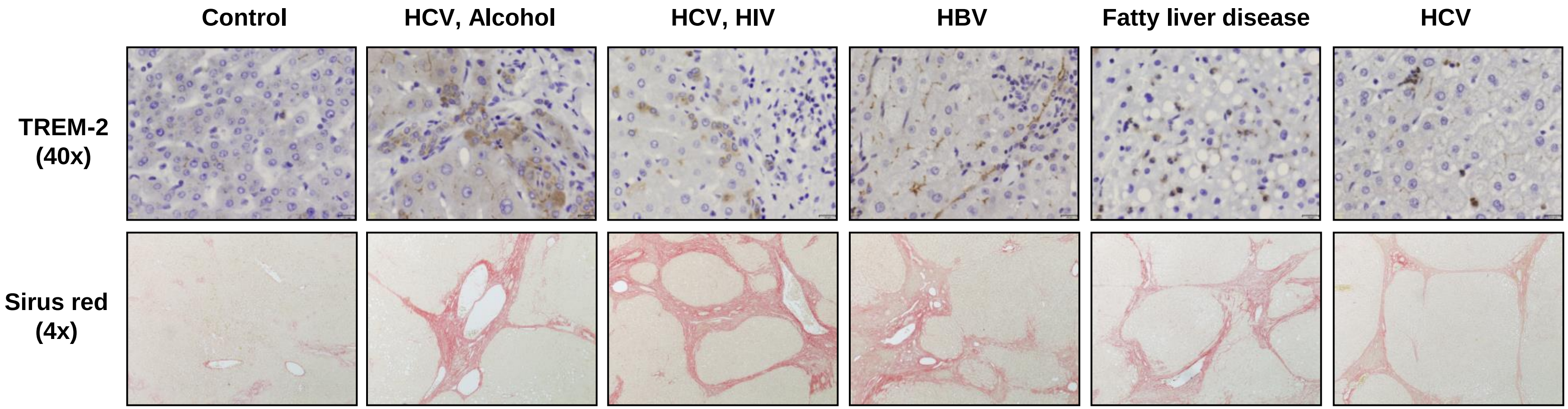

Supplementary Figure 2: Expression of *TREM-2* by immunohistochemistry during human liver disease.
TREM-2 immunohistochemistry and Sirius Red staining of a control liver and cirrhotic tissues of diverse aetiology.

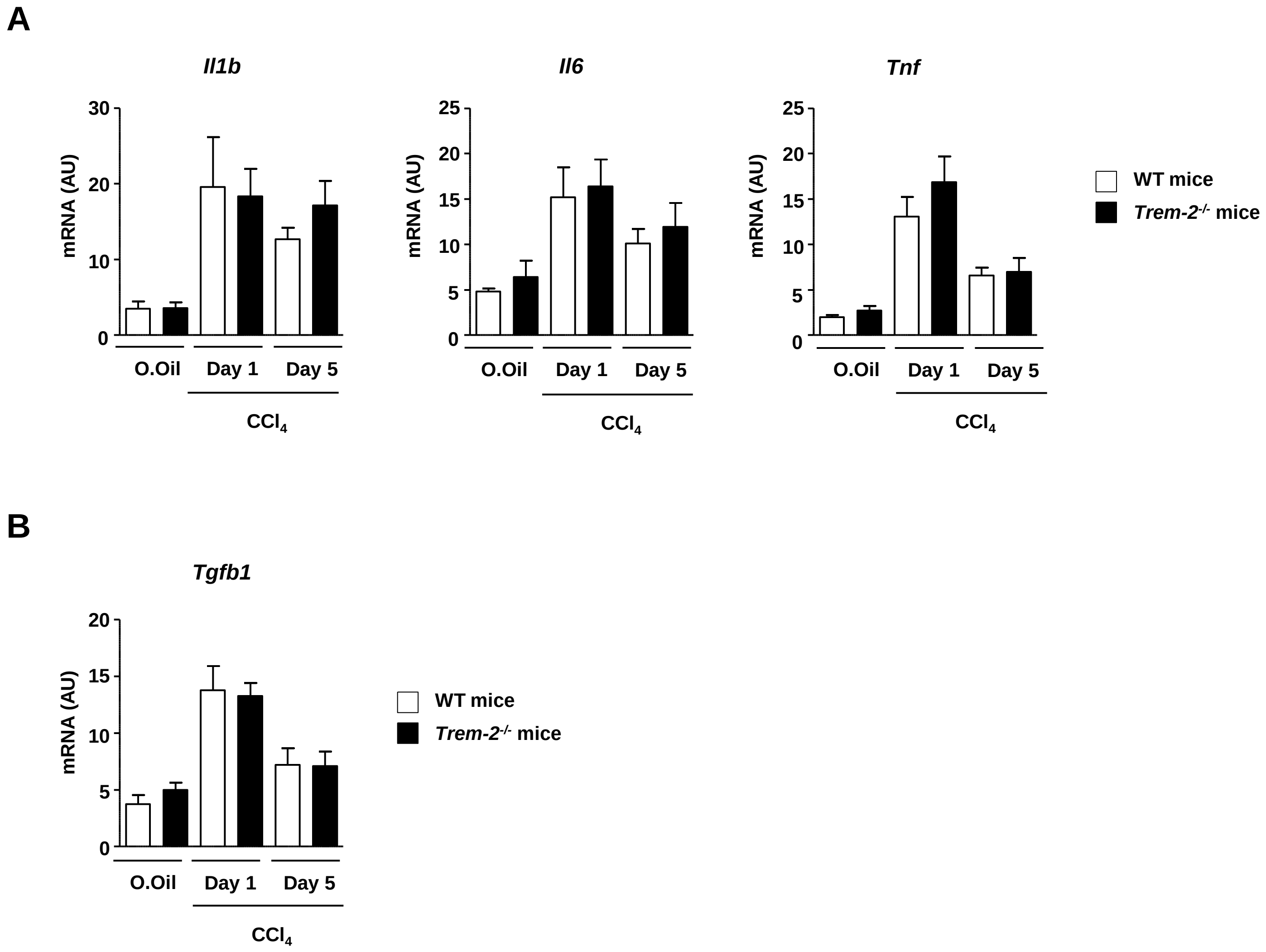

Supplementary Figure 3: No differences in *Il1b*, *Il6*, *Tnf* and *Tgfb1* between livers of WT and *Trem-2*$^{-/-}$ mice after chronic CCl_4 treatment.
(**A**) *Il1b*, *Il6* and *Tnf* cytokines and (**B**) *Tgfb1* mRNA levels following chronic CCl_4 treatment. Data represent mean ± SEM and n = 3 mice per genotype (olive oil) and 4-8 per genotype (CCl_4 both time points). The mRNA levels are expressed in arbitrary units (AU).

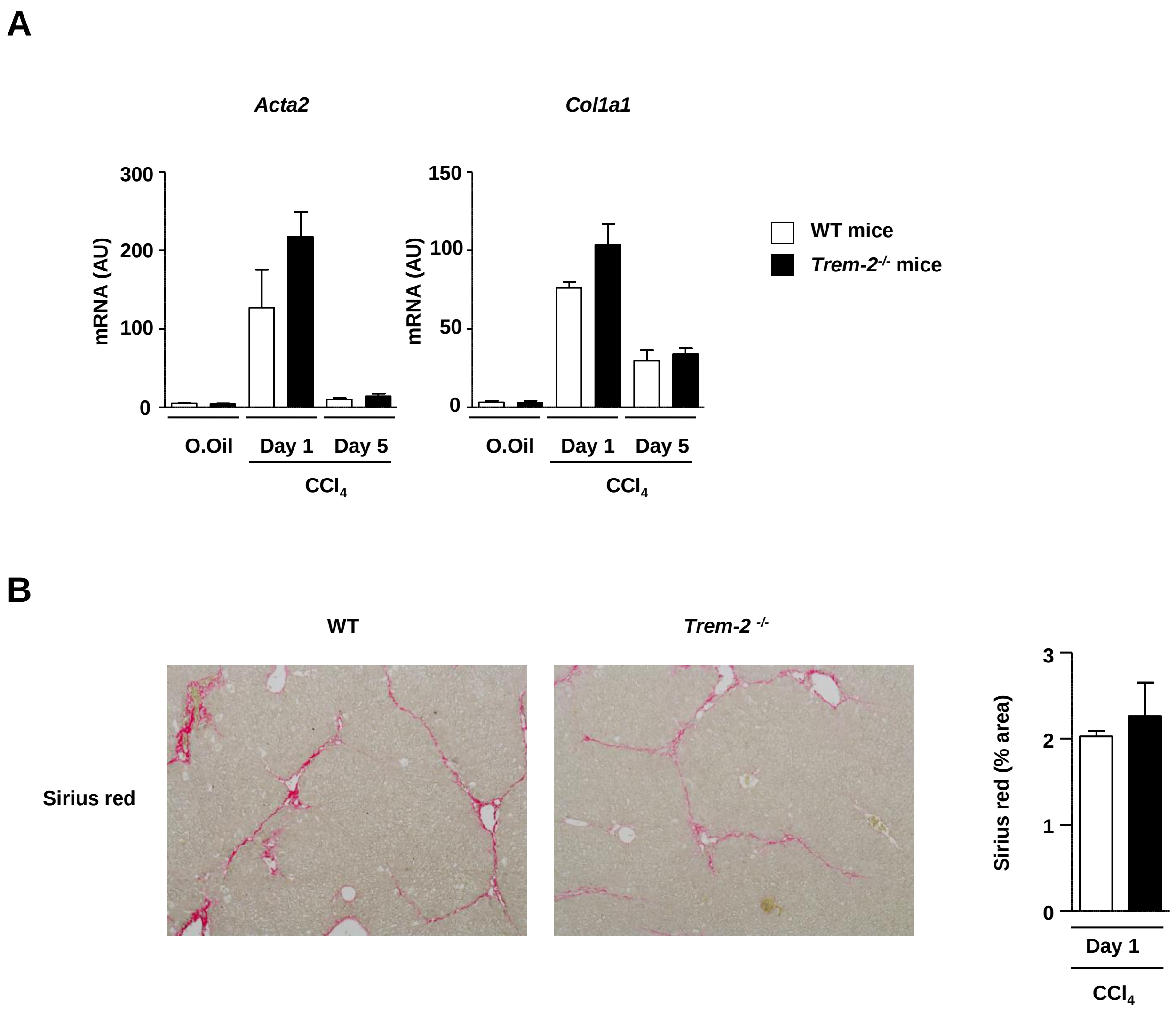

Supplementary Figure 4: TREM-2 does not impact hepatic fibrosis during chronic CCl_4.
(**A**) WT and *Trem-2*$^{-/-}$ mice were treated with CCl_4 for 8 weeks, sacrificed 1 or 5 days after the last CCl_4 injection and liver *Acta2* and *Col1a1* transcript levels were determined in WT and *Trem-2*$^{-/-}$ mice. (**B**) Representative Sirius red stained sections from day 1 are depicted. Bar chart represents quantifications of Sirius red stain and data represent mean ± SEM.

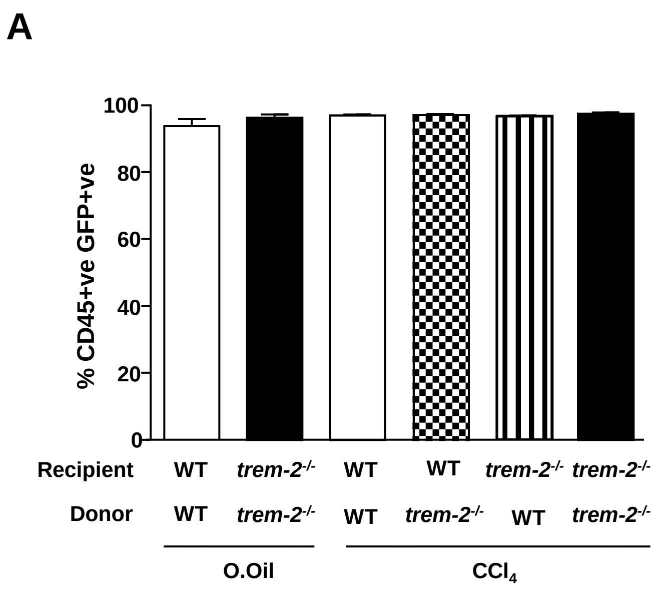

Supplementary Figure 5: Successful reconstitution of immune cells and liver neutrophil levels following bone marrow transplantation and CCl_4.

WT and *Trem-2*$^{-/-}$ mice were lethally irradiated and transplanted with WT-UbGFP$^+$ or *Trem-2*$^{-/-}$-UbGFP$^+$ bone marrow (BM) to generate WT mice (WT/WT), *Trem-2*$^{-/-}$mice (*Trem-2*$^{-/-}$/ *Trem-2*$^{-/}$) or chimeric mice (WT/ *Trem-2*$^{-/-}$) and (*Trem-2*$^{-/-}$/WT) mice (1st mouse indicates recipient and 2nd indicates donor). 8 weeks post reconstitution mice were administered CCl_4 for 8 weeks and sacrificed 1 day after the last CCl_4 injection. (**A**) Blood was isolated and flow cytometry conducted for CD45 and GFP. Depicted is the % CD45$^+$ cells that are GFP $^+$ within each group and represent mean ± SEM. n = 3 per genotype (olive oil) and 3-5 per genotype (CCl_4). Livers were isolated and (**B**) hepatic neutrophils levels were determined by gating on CD45$^+$CD11b$^+$Ly6C$^+$Ly6G$^+$GFP$^+$ cells (Supplementary fig 5). Total number of neutrophils normalised to liver weight are indicated. All data represent mean ± SEM and n = 3 per genotype (olive oil) and 3-5 per genotype (CCl_4).

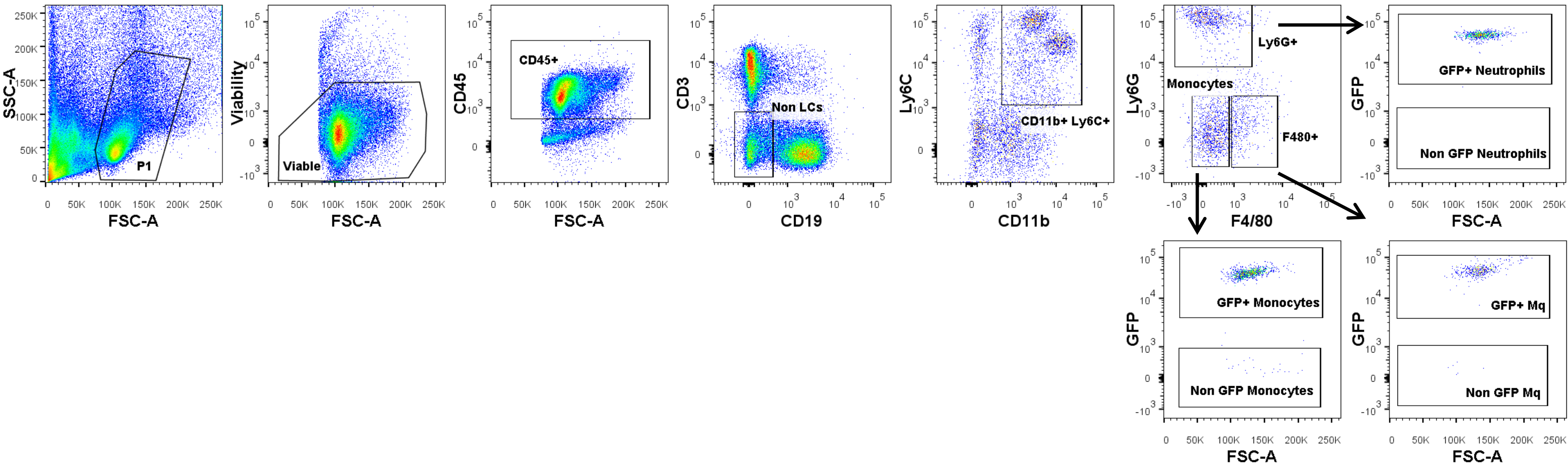

Supplementary Figure 6: Gating strategy used to determine hepatic neutrophil and macrophage numbers.
Viable cells were gated for CD45 positivity and the non-lymphocyte ($CD19^-CD3^-$) fraction was selected for being $Ly6C^+$ and $Cd11b^+$. Infiltrating neutrophils were identified as $CD45^+CD11b^+Ly6C^+Ly6G^+F4/80^-GFP^+$ cells while monocyte derived macrophages were identified as $CD45^+CD11b^+Ly6C^+Ly6G^-F4/80^+GFP^+$ cells.

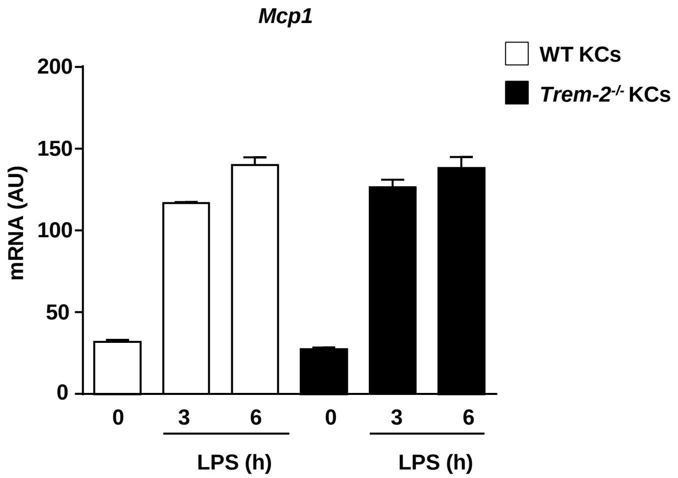

Supplementary Figure 7: KC expressed TREM-2 does not impact TLR4 driven MCP-1 production.
WT and *Trem-2*$^{-/-}$ KCs were treated with LPS (100 ng/ml) for the indicated time points (n = 4-5 per condition and time point) and levels of *Mcp1* were determined by qRT-PCR. Data represent mean ± SEM (One Way Anova, followed by Tukey´s post hoc test).

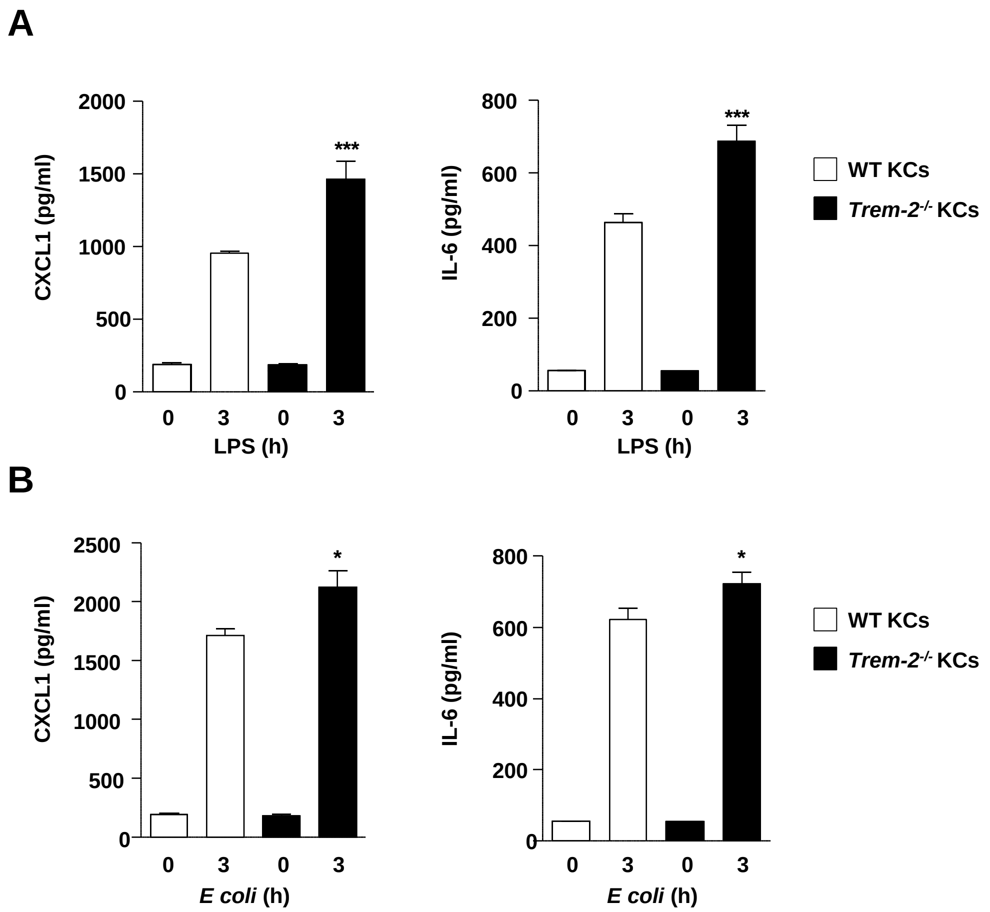

Supplementary Figure 8: Kupffer cell expressed TREM-2 dampens secreted levels of TLR4 driven CXCL1 and IL-6. WT and *Trem-2*$^{-/-}$ KCs were treated with LPS (100 ng/ml) (**A**) or (**B**) heat-killed *E. coli* (2 x 10^7 CFU/ml) for 6h (n=4-5) and levels of CXCL1 and IL-6 were evaluated in the supernatant using ELISA. Data represent mean ± SEM and $^{*, ***}$ denote a *P* value of <0.05, and <0.001 respectively versus WT (One Way Anova, followed by Tukey's post hoc test).

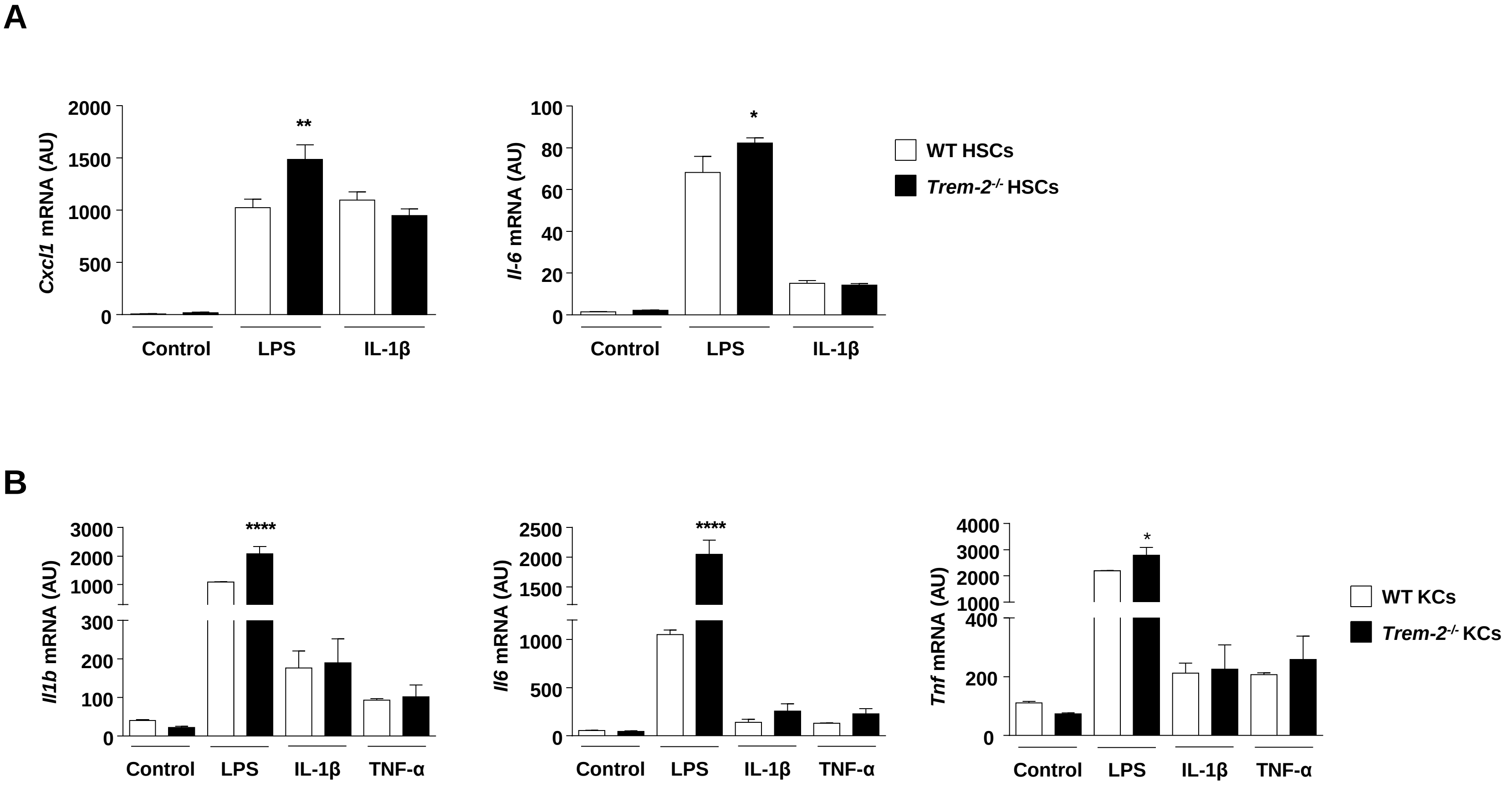

Supplementary Figure 9: LPS but not IL-1β and/or TNF-α treatment results in augmented inflammation in Trem-2$^{-/-}$ HSCs and Kupffer cells. (**A**) WT and *Trem-2*$^{-/-}$ HSCs were treated with LPS (100 ng/ml) and IL1-β (10 ng/ml) for 3h (n=4-5) and mRNA levels of *Cxcl1* and *Il-6* were evaluated. (**B**) WT and *Trem-2*$^{-/-}$ HSCs were treated with LPS (100 ng/ml), IL1-β (10 ng/ml) and TNF-α (10 ng/ml) for 3h (n=3-4) and mRNA levels of *Il6*, *Il1b* and *Tnf* were evaluated. Data represent mean ± SEM and *, **, **** denote a *P* value of <0.05, <0.01 and <0.0001 respectively versus WT (One Way Anova, followed by Tukey's post hoc test).

Supplementary Table 1:
Patient details used for TREM-2 expression in control and diseased human liver (Fig. 1A-C and Supplementary Fig. 1)

Parameters	Controls*	Cirrhotics
**Cases**, n	**21**	**23**
**Sex**, n		
Male	**16**	**22**
Female	**5**	**1**
**Age**, n (±SD)	**65,5 (±7,1)**	**60,8 (±8,7)**
**Etiology**, n		
Alcoholic	**-**	**9**
HCV	**-**	**7**
HBV	**-**	**2**
HCV, HBV	**-**	**2**
HCV, HIV	**-**	**1**
HCV, HBV, HIV	**-**	**1**
Idiopathic	**-**	**1**
**Serum ALT**, IU/L	**20,85 (±7,10)**	**54,60 (±36,82)**
**Serum AST**, IU/L	**23,52 (±5,85)**	**47,04 (±24,67)**

Data are shown as median ± SD.
HBV, hepatitis B virus; HCV, hepatitis C virus; ALT, alanine aminotransferase; AST, aspartate transaminase.
* Background normal liver from resection specimens of colorectal liver metastasis.

Supplementary Table 2:
Patient details used for Immunohistochemistry of TREM-2 in control and diseased human liver (Supplementary Fig. 2)

Parameters	Controls	Cirrhotics
**Cases**, n	**1**	**5**
**Sex**, n		
Male		**4**
Female	**1**	**1**
**Age**, n (±SD)	**49**	**58,6 (±10,6)**
**Etiology**, n		
HCV + Alcoholic	**-**	**1**
HCV + HIV	**-**	**1**
HBV	**-**	**1**
Fatty Liver disease	**-**	**1**
HCV	**-**	**1**
**Serum ALT**, IU/L	**33**	**87,8 (±51,20)**
**Serum AST**, IU/L	**32**	**97,66 (±56,36)**

Data are shown as median ± SD.
HBV, hepatitis B virus; HCV, hepatitis C virus; HIV, human immunodeficiency virus
ALT, alanine aminotransferase; AST, aspartate transaminase.

Supplementary Table 3:
Primer sequences for the different genes analysed in this study.

Gene (mouse)	Forward Primer (5´ ⟶ 3´)	Reverse Primer (5´ ⟶ 3´)
Gapdh	GCACAGTCAAGGCCGAGAAT	GCCTTCTCCATGGTGGTGAA
Acta2	TCAGCGCCTCCAGTTCCT	AAAAAAAACCACGAGTAACAAATCAA
Col1a1	TTCACCTACAGCACGCTTGTG	GATGACTGTCTTGCCCCAAGTT
Mmp13	CTTCTTCTTGTTGAGCTGGACTC	CTGTGGAGGTCACTGTAGACT
Tgfb1	CTCCCGTGGCTTCTAGTGC	GCCTTAGTTTGGACAGGATCTG
Il1b	GCAACTGTTCCTGAACTCAACT	ATCTTTTGGGGTCCGTCAACT
Il6	TAGTCCTTCCTACCCCAATTTCC	TTGGTCCTTAGCCACTCCTTC
Tnf	CCCTCACACTCAGATCATCTTCT	GCTACGACGTGGGCTACAG
Trem2	TTGCTGGAACCGTCACCATC	CACTTGGGCACCCTCGAAAC
Cxcl1	CTGGGATTCACCTCAAGAACATC	CAGGGTCAAGGCAAGCCTC
Mcp1	TTAAAAACCTGGATCGGAACCAA	GCATTAGCTTCAGATTTACGGGT
Hmox1	AAGCCGAGAATGCTGAGTTCA	GCCGTGTAGATATGGTACAAGGA
Cybb	TGTGGTTGGGGCTGAATGTC	CTGAGAAAGGAGAGCAGATTTCG
Nos2	GTTCTCAGCCCAACAATACAAGA	GTGGACGGGTCGATGTCAC
Hspa1b	GAGATCGACTCTCTGTTCGAGG	GCCCGTTGAAGAAGTCCTG
Bcl2	GTCGCTACCGTCGTGACTTC	CAGACATGCACCTACCCAGC
Bcl2l1	GACAAGGAGATGCAGGTATTGG	TCCCGTAGAGATCCACAAAAGT
Bax	TGAAGACAGGGGCCTTTTTG	AATTCGCCGGAGACACTCG

Gene (rat)	Forward Primer (5´ ⟶ 3´)	Reverse Primer (5´ ⟶ 3´)
Gapdh	TGTGAACGGATTTGGCCGTA	ATGAAGGGGTCGTTGATGGC
Trem2	AAGATGCTGGAGACCTCTGG	GGATGCTGGCTGTAAGAAGC

Gene (human)	Forward Primer (5´ ⟶ 3´)	Reverse Primer (5´ ⟶ 3´)
GAPDH	CCAAGGTCATCCATGACAAC	TGTCATACCAGGAAATGAGC
TREM2	ACGAGATCTTGCACAAGGCA	GGTAGAGACCCGCATCATGG
COL1A1	GATGGCTGCACGAGTCACAC	AACGTCGAAGCCGAATTCCT
IL1B	AGCTACGAATCTCCGACCAC	CGTTATCCCATGTGTCGAAGAA
IL6	AAAGAGGCACTGGCAGAAAA	AGCTCTGGCTTGTTCCTCAC
IL8	GTGCAGTTTTGCCAAGGAGT	ACTTGTCCACAACCCTCTGC
MCP1	CAGCCAGATGCAATCAATGCC	TGGAATCCTGAACCCACTTCT

Primer sequences used to detect human TREM-2 overexpression in LX2 HSCs.

Gene (human)	Forward Primer (5´ ⟶ 3´)	Reverse Primer (5´ ⟶ 3´)
TREM2	CAAGATTCTAGCAGCCAGCG	CTCAGCCCTGGCAGAGTTTG
