# Supplementary material for: Non-parenchymal TREM-2 protects the liver from immune-mediated hepatocellular damage
Source: Gut. 2018 Jan 27;68(3):533–46. doi: 10.1136/gutjnl-2017-314107 (PMC6580759; doi:10.1136/gutjnl-2017-314107)
Supplement: Supplementary file 2 [file gutjnl-2017-314107supp002.pdf]

Non-Parenchymal TREM-2 protects the Liver from Immune-mediated Hepatocellular damage

Maria J Perugorria[1,2,3], Aitor Esparza-Baquer[2], Fiona Oakley[1], , Ibone Labiano[2], Ana Korosec[4,5], Alexander Jais[6*], Jelena Mann[1], Dina Tiniakos[1], Santos-Laso A[2], Arbelaiz A[2], Riem Gawish[4,5#], Sampedro A[7], Fontanellas A[7], Elizabeth Hijona[2], Raúl Jiménez-Agüero[2], Harald Esterbauer[6], Dagmar Stoiber[8,9], Luis Bujanda[2], Jesús M. Banales[2,3], Sylvia Knapp[4,5], Omar Sharif[4,5,8§] and Derek A Mann[1]

[1]Institute of Cellular Medicine, Faculty of Medical Sciences, 4th Floor, William Leech Building, Newcastle University, Framlington Place, Newcastle upon Tyne, NE2 4HH, UK. [2]Department of Liver and Gastrointestinal Diseases, Biodonostia Research Institute, Donostia University Hospital, University of the Basque Country (UPV-EHU), CIBERehd, San Sebastian, Spain. [3]IKERBASQUE, Basque Foundation for Science, Bilbao, Spain. [4]Research Center for Molecular Medicine of the Austrian Academy of Sciences, 1090 Vienna, Austria. [5]Department of Medicine I, Laboratory of Infection Biology, Medical University of Vienna, Austria. [6]Department of Laboratory Medicine, Medical University of Vienna, Austria. [7]CIMA-University of Navarra, Gene Therapy and Hepatology Area, Pamplona, Spain. [8]Ludwig Boltzmann Institute for Cancer Research, Vienna, Austria. [9]Institute of Pharmacology, Center of Physiology and Pharmacology, Medical University of Vienna, Vienna, Austria

Authorship note: Derek A Mann and Omar Sharif are co-senior authors and contributed equally.

***** current affiliation (AJ): Max Planck Institute for Metabolism Research, Cologne, Germany

[#] current affiliation (RG): Institute of Animal Breeding and Genetics, Department of Biomedical Science, University of Veterinary Medicine Vienna, Austria

[§] current affiliation (OS): Ludwig Boltzmann Institute of Cancer Research, Vienna, Austria

MATERIAL AND METHODS

Bone marrow transplantation

8-10 week-old male WT and *Trem-2*$^{-/-}$ mice were irradiated by a single administration of γ irradiation (9 Gy). Bone marrow was isolated from *Trem-2*$^{-/-}$/Ub-GFP or WT-GFP expressing mice and irradiated mice were immediately reconstituted by intravenous injection of 2×10^6 freshly isolated bone marrow cells to generate either WT mice (WT/WT), *Trem-2*$^{-/-}$ mice (*Trem-2*$^{-/-}$/*Trem-2*$^{-/-}$) or chimeric mice (WT/*Trem-2*$^{-/-}$) and (*Trem-2*$^{-/-}$/WT) mice (1st mouse indicates recipient and 2nd indicates donor). One mouse of each genotype was left without reconstitution to control for efficiency of irradiation. Mice were kept under sterile conditions for 8 weeks to allow for reconstitution following which repetitive CCl_4 injury was performed. Bone marrow reconstitution was confirmed by flow cytometry for GFP^+ blood leukocytes (Supplemental Figure 5).

Acute CCl_4 treatment after gut sterilization with antibiotics

WT and *Trem-2*$^{-/-}$ male mice that were born and weaned at the Biodonostia Research Institute were treated with a combination of antibiotics; ampicillin (Sigma Aldrich; 1 g/l), neomycin (GIBCO: 1 g/l), metronidazole (Acros Organics; 1 g/l) and vancomycin (Pfizer; 500mg/l) in drinking water at 6 weeks of age. After 4 weeks of antibiotic treatment, mice were intraperitoneally injected with CCl_4 at 2 µl/g body weight (CCl_4: olive oil at 1:1 [vol:vol]) and sacrificed after 12h. Four hours before sacrifice, all animals were gavaged with 4kDa FITC-dextran (Sigma Aldrich, 80mg/100g body weight) to assess intestinal permeability as previously described.[1] FITC-dextran measurements were performed in serum by fluorometry.

Isolation of mouse hepatocytes, KCs, HSCs, BMDMs and treatments

Hepatocytes, KCs and HSCs were isolated from mice as described.[2] BMDM were isolated by flushing the femurs and tibias of mice in RPMI 1640 and differentiated in RPMI 1640 supplemented with 1% pen/strep, 10% FCS and 10% L-929 conditioned medium for 7 days. Activated HSCs from rat livers injured by bile duct ligation (BDL) for 10 days or chronically injured with CCl_4 for 3 weeks were isolated as previously described.[3] Where indicated cells were plated at 10^6/ml in 6 well dishes in RPMI supplemented with 10% heat-inactivated FCS and 1% pen/strep and stimulated with 2 x 10^7/ml heat-killed *E. coli* (O18:K1), 100 ng/ml LPS (Sigma Aldrich), 10 ng/ml IL-1β (Peprotech) or 10 ng/ml TNF-α (Peprotech) for various times. Following cell treatments, cells were processed for Western blot, RNA extraction or supernatant was harvested and stored at -20ºC for cytokine measurements using ELISA.

Hepatocyte cell viability assay

Hepatocytes isolated from WT and *Trem-2*$^{-/-}$ mice were plated at 10^4 cells/well into 96 well dishes in William´s E Medium supplemented with 10% FBS, 1% pen/strep and 1% NEAA. After 3h, medium was changed to William´s E Medium supplemented with 0.5% FBS, 1% pen/strep and 1% NEAA containing 5 mM or 10 mM APAP (Sigma Aldrich). Hepatocytes were treated with APAP for 24 hours after which the CellTiter-Glo® Luminescent Cell Viability Assay (Promega) was carried out according to the manufacturer´s instructions.

Isolation of liver cells and flow cytometry

Liver mononuclear cells were isolated as previously described.[4] Post injury, mice were sacrificed and the livers were perfused via the portal vein with HBSS, cut into small pieces and digested for 1h at 37ºC in RPMI 1640 containing 0.05% collagenase/Dispase (Roche) and 0.01% trypsin inhibitor (GIBCO). The liver suspension was pressed through a 40 µM cell strainer, centrifuged at 800g for 10 min at 4ºC and the cell pellet was resuspended in 10 ml RPMI 1640. Cell suspensions were overlayed onto 15 ml of 33% (vov/vol) Percoll solution (Sigma, P1644) and centrifuged for 30 min at room temperature with no brake. The supernatant was removed and erythrocytes were lysed by resuspending the pellet in 2 ml of red blood cell lysis buffer for 4 min, after which 8 ml of RPMI1640 medium was added. Cell suspensions were spun at 800g for 5-10 min at 4ºC, supernatant was removed and the cells were washed twice with 10 ml RPMI1640 medium. Cells were resuspended in PBS/2% FCS and total cell numbers per liver were enumerated using a hemocytometer (Turck chamber). Subsequently, Fc receptors were blocked with CD16/CD32 antibody (eBioscience, clone 93) and mononuclear cells were stained with; Fixable Viability Dye eFluor 780 (eBioscience, Cat no 65-0865), CD45 V500 (BD Horizon, clone 30-F11), Ly6G PE, Ly6C BV570 (both from BioLegend, clone 1A8 and HK1.4 respectively) and F4/80 PerCP/Cy5.5, CD11b AF700 (both from eBioscience, clone BM8 and M1/70 respectively). After a washing step, cells were resuspended in PBS and analyzed by flow cytometry (BD LSR Fortessa). After excluding doublets, gating was performed only on viable cells. Neutrophils were identified as $CD45^{+}CD11b^{+}Ly6C^{+}Ly6G^{+}$, $F4/80^{-}$ cells. Macrophages were identified as $CD45^{+}CD11b^{+}Ly6C^{+}Ly6G^{-}F4/80^{+}$ cells. The number of cells per

liver was normalised to the liver weight and the total cell count. Blood was stained by directly adding antibodies for 30 min to 50 µl of sample. Cells were lysed by adding 140 µL ADG lysis buffer (Andergrub Bioresearch) for 10 min, after which 3.5 ml H_2O was added for 7 min. Samples were washed twice with PBS/FCS and analyzed by flow cytometry (BD LSR Fortessa).

Hepatocytes, KCs and HSCs were isolated and flow cytometry analysis on single liver cell suspensions was performed according to standard procedures. The following antibodies were used: Monoclonal rat anti-mouse TREM-2 APC (FAB17291A) (R&D) and isotype control Rat IgG2B APC labeled (IC013A) (R&D).

Human hepatic myofibroblasts (HM) were isolated from livers of adult male patients after surgical resection, subject to patient consent (as approved by the Newcastle and North Tyneside Local Research Ethics Committee, (10/H0906/41)) and were cultured in DMEM supplemented with 100 U/mL penicillin, 100 µg/mL streptomycin, 2 mM L-glutamine, and 16% fetal bovine serum and maintained at 37°C at an atmosphere of 5% CO_2.

TREM-2 overexpression in human hepatic LX-2 cells

LX-2 cells were plated into 6 well dishes ($3x10^5$ cells/well) in DMEM containing 2% (v/v) FBS. After 24h, human TREM-2 expression plasmid or pCMV3-untagged negative control vector (Sino Biological Inc, Cat no HG11084-UT or CV011 respectively) were transfected into LX-2 cells with Lipofectamine 2000 reagent (Invitrogen) with the ratio of DNA (µg) to lipofectamine (µl) at 1:1, according to the manufacturer's instructions. Post transfection medium was removed 6h later and replaced by DMEM 2% (v/v) FBS with antibiotics. Twenty

four hours after transfection, cells were treated with 100 ng/ml LPS (Sigma Aldrich) for 3h.

Immunohistochemistry and image analysis

Immunostaining was performed on formalin-fixed, paraffin-embedded mouse and human liver tissue sections (3 controls and 5 cirrhotic cases of variable etiology as described in Supplemental Table 2). 4-HNE, Ly6G and F4/80 immunostaining of mouse liver and TREM-2 immunostaining of human liver were conducted by deparaffinising sections in xylene and rehydrating in graded ethanol followed by blocking endogenous peroxidase in 3.5% H_2O_2 in PBS for 10 min. Thereafter, sections were subjected to antigen-retrieval using citrate buffer pH6.0 (Vector laboratories) and those slides where human TREM-2 or mouse 4-HNE immunostaining was evaluated were blocked in 10% goat serum in PBS (Vector laboratories), while slides stained for Ly6G or F4/80 were blocked in 10% rabbit serum in PBS (Vector laboratories) for 10 min. Slides were subsequently incubated with rabbit polyclonal anti-human TREM-2 (Sigma HPA010917) diluted 1:200, rabbit polyclonal anti-mouse 4-HNE (AbCAM, ab46545) diluted 1:200, rat monoclonal anti-mouse Ly6G (BD Pharmingen 551459) diluted 1:70 and rat monoclonal anti-mouse F4/80 (Serotec MCA497G) diluted 1:100 in the respective blocking agents for 1h. Avidin sites were blocked using the avidin-biotin blocking kit (Vector laboratories). Sections were washed, and incubated with biotinylated anti-rabbit antibody (Vector Laboratories BA-1000) diluted 1:200 in 10% goat serum/PBS or biotinylated anti-rat antibody (Vector Laboratories BA-4001) diluted 1:200 in 10% rabbit serum for 30 min at room temperature. Binding was visualized using the Vectastain ABC kit (Vector

laboratories) followed by incubation with 3,3-diaminobenzidine (DAB) conjugated to HRP (Vector laboratories). Sections were lightly counter stained with haematoxylin (Mayer, Merck), dehydrated, coverslipped and subjected to light microscopy. We performed NIMP immunostaining as previously described.[2] F4/80 and Ly6G levels were evaluated by manual counts on eight random fields per mouse. Evaluation of immunohistochemistry for human TREM-2 was performed by an experienced hepatopathologist (DGT) who recorded the type and topography of immunostained cells and evaluated the intensity of TREM-2 specific cytoplasmic immunostaining as mild, moderate or severe compared to the immunostaining intensity of neutrophils in control liver sections. An isotype control was used to verify the specificity of the TREM-2 antibody on human spleen sections.

Liver histology and scoring

Liver tissue samples were routinely formalin-fixed and paraffin-embedded. Five μm-thick sections were cut and stained with haematoxylin & eosin for morphological assessment of necroinflammation, hepatocyte injury, and steatosis, and with Sirius red histochemical stain to evaluate fibrosis. All sections were blindly assessed by an experienced hepatopathologist (DGT).

Semi-quantitative evaluation of inflammation severity (scored 0-3, 0=none, 1=mild, 2=moderate, 3=severe) and extent of necrosis (score 0-3, 0=none, 1=focal, 2=moderate, 3=extensive) was undertaken. Acinar topography, type of inflammatory cells involved, and type of necrosis (zone 3, central-central bridging necrosis) were recorded. Hepatocyte injury, in the form ballooned hepatocytes and apoptotic (oxyphilic) bodies, was also semi-quantitatively

evaluated (score 0-3, 0=none, 1=few, 2=several, 3=many), and acinar involvement (zone 3 or both zones 3 and 2) was recorded. The presence, type and topography of hepatocyte steatosis was recorded.

The extent of fibrosis was evaluated using a 7-tier staging system (0-6) based on the characteristic topography of fibrosis in chronic CCl_4-related liver damage (0 = no significant fibrosis, 1 = mild zone 3 sinusoidal fibrosis-Z3SF, 2 = prominent z3SF, 3=Z3SF plus central-central (C-C) fibrous septa, 4=advanced bridging fibrosis with C-C and few central-portal fibrous septa, 5=as per 4 plus occasional fully circumscribed nodules, 6=cirrhosis).

SDS-PAGE and immunoblotting

Proteins were fractionated by 12.5% SDS-PAGE and then transferred onto nitrocellulose membrane. Blots were blocked in Tris-buffered saline and Tween-20 (0.1%) (T-TBS) containing 5% BSA or 5% milk protein before incubation overnight at 4ºC with primary antibodies at 1:1000 dilution. Primary antibodies used were: phospho-p44/42 MAPK (Erk1/2) (Thr202/Tyr204) (9101), p44/42 MAPK (Erk1/2) (9102), phospho-p38 MAPK (Thr180/Tyr182) (9211), p38 MAPK (9212), phospho-SAPK/JNK (Thr183/Tyr185) (9251), SAPK/JNK (9252), phospho-NFκB p65 (Ser536) (3033) (Cell Signaling), NFκB p65 (F-6) (sc-8008), IκB-α (C-21) (sc-371) (Santa Cruz Biotechnology), β-Actin (A5316) (Sigma). Membranes were washed in T-TBS and incubated with secondary anti-rabbit IgG (7074) (Cell Signaling) or anti-mouse IgG (A4416) (Sigma) HRP-conjugated antibodies at 1:5000 dilution for 1h. Blots were washed and antigen was detected by ECL (Amersham Biosciences).

ROS detection and oxygen consumption assays

Intracellular reactive oxygen species (ROS) levels were measured using the fluoroprobe dihydrorhodamine 123 (DHR123). BMDM were plated at 10^6 cells/ml in 24 well dishes and treated with 100 ng/ml LPS for 3h. Following cell treatment, 10 µM dihydrorhodamine 123 (DHR123, Invitrogen) was added to the wells for 10 min and the cells were washed twice with PBS, resuspended in PBS and analysed using flow cytometry. Analysis of oxygen consumption rates (OCR) was performed using the XF24 Flux Analyzer (Seahorse Bioscience). BMDM were seeded into XF 24-well cell culture microplates and allowed to recover for 24h, after which they were treated with 100 ng/ml LPS for 3h, supernatant was removed and 600 µl of buffer free Assay Medium (Seahorse Bioscience) was added to each well before transferring the cells to a CO_2 free incubator and maintaining them at 37°C for 1h prior to the assay. Following instrument calibration, cells were transferred to the XF24 Flux Analyzer to record cellular OCR. The measurement protocol consisted of 2 min mixture, 2 min wait and 4 min OCR measurement times. For the mitochondrial stress test, ATP synthase was inhibited by injection of 1 µM oligomycin, mitochondrial uncoupling was achieved with 3 µM FCCP and electron transport was shut down by rotenone/antimycin injection (1 µM each). Maximal respiration was determined as the OCR values following FCCP but prior to rotenone/antimycin injection.

Cytokine and chemokine detection by ELISA

KCs and activated HSCs were plated at 10^6 cells/ml in 6 well dishes in RPMI supplemented with 10% heat-inactivated FCS and 1% pen/strep and stimulated

with $2x10^7$/ml heat-killed *E. coli* (O18:K1) or 100ng/ml LPS for various times. Following cell treatments, supernatant was harvested and stored at -20ºC until ELISA was performed. IL-6, CXCL1 and MCP-1 were measured using specific ELISAs according to the manufacturer’s instructions (R&D Systems).

Quantification of 4-HNE levels in liver tissue

4-hydroxynonenal (4-HNE) protein adducts were measured in liver tissues of mice using the OxiSelect™ HNE Adduct Competitive ELISA (Cell Biolabs, San Diego, CA) according to manufacturer´s instructions.

RNA isolation and Quantitative RT-PCR analysis

RNA isolation and qRT-PCR were performed as previously described.[2] Primer sequences are included in Supplemental Table 3. Gene expression was determined using the ΔCT calculation and mRNA levels are expressed as arbitrary units (AU).

Statistical analysis

Statistical analysis was performed with the Graph Pad Prism version 6.00 software (Graph Pad Software, San Diego, CA). Data are expressed as mean ± standard error of the mean (SEM). Data were compared among groups using a Student T test in case the data followed a normal distribution. In case the data did not follow a normal distribution a Mann Whitney Test was used. One-way analysis of variance with Tukey’s post hoc test for individual subgroup comparison was used when more than two groups were compared. Statistically

significant data is represented in figures where *, **, ***, **** denote a P value of <0.05, <0.01, < 0.001 and < 0.0001 respectively.

SUPPLEMENTARY FIGURE LEGENDS:

Supplementary Figure 1: Correlation of TREM-2 levels with AST and pro-inflammatory cytokines.

qRT-PCR analysis of TREM-2 in control human liver and cirrhotic samples was performed and correlated with (A) AST and (B) proinflammatory cytokine expression (*IL-6*, *IL-8* and *IL-1B*). n = 21 control cases and 23 cirrhotic livers.

Supplementary Figure 2: Expression of TREM-2 by immunohistochemistry during human liver disease.

TREM-2 immunohistochemistry and Sirius Red staining of a control liver and cirrhotic tissues of diverse aetiology.

Supplementary Figure 3: No differences in *Il1b*, *Il6*, *Tnf* and *Tgfb1* between livers of WT and *Trem-2*$^{-/-}$ mice after chronic CCl_4 treatment.

(**A**) *Il1b*, *Il6* and *Tnf* cytokines and (**B**) *Tgfb1* mRNA levels following chronic CCl_4 treatment. Data represent mean ± SEM and n = 3 mice per genotype (olive oil) and 4-8 per genotype (CCl_4 both time points). The mRNA levels are expressed in arbitrary units (AU).

Supplementary Figure 4: TREM-2 does not impact hepatic fibrosis during chronic CCl_4.

(**A**) WT and *Trem-2*$^{-/-}$ mice were treated with CCl_4 for 8 weeks, sacrificed 1 or 5 days after the last CCl_4 injection and liver *Acta2* and *Col1a1* transcript levels were determined in WT and *Trem-2*$^{-/-}$ mice. (**B**) Representative Sirius red

stained sections from day 1 are depicted. Bar chart represents quantifications of Sirius red stain and data represent mean ± SEM.

Supplementary Figure 5: Successful reconstitution of immune cells and liver neutrophil levels following bone marrow transplantation and CCl_4.

WT and *Trem-2*$^{-/-}$ mice were lethally irradiated and transplanted with WT-UbGFP^{+} or *Trem-2*$^{-/-}$-UbGFP^{+} bone marrow (BM) to generate WT mice (WT/WT), *Trem-2*$^{-/-}$mice (*Trem-2*$^{-/-}$/ *Trem-2*$^{-/}$) or chimeric mice (WT/*Trem-2*$^{-/-}$) and (*Trem-2*$^{-/-}$/WT) mice (1st mouse indicates recipient and 2nd indicates donor). Eight weeks post reconstitution mice were administered CCl_4 for 8 weeks and sacrificed 1 day after the last CCl_4 injection. (**A**) Blood was isolated and flow cytometry conducted for CD45 and GFP. Depicted is the % CD45^{+} cells that are GFP $^{+}$ within each group and represent mean ± SEM. n = 3 per genotype (olive oil) and 3-5 per genotype (CCl_4). Livers were isolated and (**B**) hepatic neutrophils levels were determined by gating on CD45^{+}CD11b^{+}Ly6C^{+}Ly6G^{+}GFP^{+} cells (**Supplementary Fig 6**). Total number of neutrophils normalised to liver weight are indicated. All data represent mean ± SEM and n = 3 per genotype (olive oil) and 3-5 per genotype (CCl_4).

Supplementary Figure 6: Gating strategy used to determine hepatic neutrophil and macrophage numbers.

Viable cells were gated for CD45 positivity and the non-lymphocyte (CD19^{-}CD3^{-}) fraction was selected for being Ly6C^{+} and Cd11b^{+}. Infiltrating neutrophils were identified as CD45^{+}CD11b^{+}Ly6C^{+}Ly6G^{+}F4/80^{-}GFP^{+} cells while monocyte

derived macrophages were identified as $CD45^+CD11b^+Ly6C^+Ly6G^-F4/80^+GFP^+$ cells.

Supplementary Figure 7: KC expressed TREM-2 does not impact TLR4 driven MCP-1 production.

WT and *Trem-2*$^{-/-}$ KCs were treated with LPS (100 ng/ml) for the indicated time points (n=4-5 per condition and time point) and levels of *Mcp1* were determined by qRT-PCR. Data represent mean ± SEM (One Way Anova, followed by Tukey´s post hoc test).

Supplementary Figure 8: KC expressed TREM-2 dampens secreted levels of TLR4 driven CXCL1 and IL-6.

WT and *Trem-2*$^{-/-}$ KCs were treated with LPS (100 ng/ml) (**A**) or (**B**) heat-killed *E. coli* (2 x 10^7 CFU/ml) for 6h (n=4-5) and levels of CXCL1 and IL-6 were evaluated in the supernatant using ELISA. Data represent mean ± SEM and $^{*, ***}$ denote a *P* value of <0.05, and <0.001 respectively versus WT (One Way Anova, followed by Tukey's post hoc test).

Supplementary Figure 9: LPS but not IL-1β and/or TNF-α treatment results in augmented inflammation in *Trem-2*$^{-/-}$ HSCs and Kupffer cells.

(A) WT and *Trem-2*$^{-/-}$ HSCs were treated with LPS (100 ng/ml) and IL1-β (10 ng/ml) for 3h (n = 4-5) and mRNA levels of *Cxcl1* and *Il-6* were evaluated. (B) WT and *Trem-2*$^{-/-}$ KCs were treated with LPS (100 ng/ml), IL1-β (10 ng/ml) and TNF-α (10 ng/ml) for 3h (n = 3-4) and mRNA levels of *Il-6, Il-1β* and *Tnf-α* were evaluated. Data represent mean ± SEM and *, **, **** denote a P value of

<0.05, <0.01 and <0.0001 respectively versus WT (One Way Anova, followed by Tukey’s post hoc test).

Supplementary Table 1:

Patient details used for *TREM-2* expression in control and diseased human liver (Fig. 1A-C and Supplementary Fig. 1)

Supplementary Table 2:

Patient details used for immunohistochemistry of TREM-2 in control and diseased human liver (Supplementary Fig. 2)

Supplementary Table 3:

Primer sequences for the different genes analysed in this study.

REFERENCES

1. Fouts DE, Torralba M, Nelson KE, Brenner DA, Schnabl B. Bacterial translocation and changes in the intestinal microbiome in mouse models of liver disease. *Journal of hepatology* 2012;56(6):1283-92.
2. Perugorria MJ, Murphy LB, Fullard N, Chakraborty JB, Vyrla D, Wilson CL, et al. Tumor progression locus 2/Cot is required for activation of extracellular regulated kinase in liver injury and toll-like receptor-induced TIMP-1 gene transcription in hepatic stellate cells in mice. *Hepatology* 2013;57(3):1238-49.
3. Mann J, Chu DC, Maxwell A, Oakley F, Zhu NL, Tsukamoto H, et al. MeCP2 controls an epigenetic pathway that promotes myofibroblast transdifferentiation and fibrosis. *Gastroenterology* 2010;138(2):705-14, 14 e1-4.
4. Watarai H, Nakagawa R, Omori-Miyake M, Dashtsoodol N, Taniguchi M. Methods for detection, isolation and culture of mouse and human invariant NKT cells. *Nat Protoc* 2008;3(1):70-8.
